# Synergic Effects of Temperature and Irradiance on the Physiology of the Marine *Synechococcus* Strain WH7803

**DOI:** 10.3389/fmicb.2020.01707

**Published:** 2020-07-24

**Authors:** Ulysse Guyet, Ngoc A. Nguyen, Hugo Doré, Julie Haguait, Justine Pittera, Maël Conan, Morgane Ratin, Erwan Corre, Gildas Le Corguillé, Loraine Brillet-Guéguen, Mark Hoebeke, Christophe Six, Claudia Steglich, Anne Siegel, Damien Eveillard, Frédéric Partensky, Laurence Garczarek

**Affiliations:** ^1^CNRS, UMR 7144 Adaptation and Diversity in the Marine Environment, Station Biologique de Roscoff, Sorbonne Université, Roscoff, France; ^2^LS2N, UMR CNRS 6004, IMT Atlantique, ECN, Université de Nantes, Nantes, France; ^3^DYLISS (INRIA–IRISA)–INRIA, CNRS UMR 6074, Université de Rennes 1, Rennes, France; ^4^CNRS, FR2424, ABiMS, Station Biologique, Sorbonne Université, Roscoff, France; ^5^CNRS, UMR 8227 Integrative Biology of Marine Models (LBI2M), Station Biologique de Roscoff, Sorbonne Université, Roscoff, France; ^6^Faculty of Biology, University of Freiburg, Freiburg, Germany

**Keywords:** marine cyanobacteria, *Synechococcus*, transcriptomics, light stress, temperature stress, UV radiations

## Abstract

Understanding how microorganisms adjust their metabolism to maintain their ability to cope with short-term environmental variations constitutes one of the major current challenges in microbial ecology. Here, the best physiologically characterized marine *Synechococcus* strain, WH7803, was exposed to modulated light/dark cycles or acclimated to continuous high-light (HL) or low-light (LL), then shifted to various stress conditions, including low (LT) or high temperature (HT), HL and ultraviolet (UV) radiations. Physiological responses were analyzed by measuring time courses of photosystem (PS) II quantum yield, PSII repair rate, pigment ratios and global changes in gene expression. Previously published membrane lipid composition were also used for correlation analyses. These data revealed that cells previously acclimated to HL are better prepared than LL-acclimated cells to sustain an additional light or UV stress, but not a LT stress. Indeed, LT seems to induce a synergic effect with the HL treatment, as previously observed with oxidative stress. While all tested shift conditions induced the downregulation of many photosynthetic genes, notably those encoding PSI, cytochrome *b_6_/f* and phycobilisomes, UV stress proved to be more deleterious for PSII than the other treatments, and full recovery of damaged PSII from UV stress seemed to involve the neo-synthesis of a fairly large number of PSII subunits and not just the reassembly of pre-existing subunits after D1 replacement. In contrast, genes involved in glycogen degradation and carotenoid biosynthesis pathways were more particularly upregulated in response to LT. Altogether, these experiments allowed us to identify responses common to all stresses and those more specific to a given stress, thus highlighting genes potentially involved in niche acclimation of a key member of marine ecosystems. Our data also revealed important specific features of the stress responses compared to model freshwater cyanobacteria.

## Introduction

All microorganisms are constrained to adjust their metabolism in order to maintain their ability to survive in constantly changing environments. These variations can occur at different timescales, from seconds to several cell generations. While long-term variations often induce adaptation, whereby the natural selection alters the genetic composition (e.g., by gene loss/gain or substitutions; Galhardo et al., 2007; Doré et al., under revision), short-term variations of the environment can be managed by phenotypic plasticity, involving physiological adjustments that allow to maintain cellular performance across varying environmental conditions (Dudley, 2004; Gabriel, 2005). Bacteria possess numerous mechanisms that enable acclimation to the variety of possible external stresses (Schimel et al., 2007). These range from up or downregulation of a single metabolic process to the activation of complex gene expression networks, e.g., through regulation by two-component sensory systems that translate extracellular signals into intracellular responses (Suzuki et al., 2001; Los et al., 2010). In this context, comparing global gene expression profiles on laboratory strains submitted to different individual stresses and for which conditions can be perfectly controlled, allows not only to uncover stress-specific global transcriptional responses but also to shed light on novel or unsuspected cellular stress responses.

Most transcriptomic studies so far have focused on model microorganisms, such as *Escherichia coli* (Kannan et al., 2008; Wang et al., 2009; Jozefczuk et al., 2010), *Bacillus subtilis* (Mostertz et al., 2004; Nicolas et al., 2012), *Caulobacter crescentus* (da Silva Neto et al., 2013), or *Chlamydomonas reinhardtii* (Nguyen et al., 2008). In the specific case of cyanobacteria, changes in whole transcriptomes have been extensively investigated in the unicellular freshwater strains *Synechocystis* sp. PCC 6803 and *Synechococcus* sp. PCC 7942 in response to various environmental conditions including light (Hihara et al., 2001; Huang et al., 2002), temperature (Suzuki et al., 2001; Mikami et al., 2002; Inaba et al., 2003), oxidative stress (Hihara et al., 2003; Singh et al., 2004), nutrient stresses (Singh et al., 2003; Suzuki et al., 2004; Krasikov et al., 2012; Blanco-Ameijeiras et al., 2017) and/or change in ambient CO_2_ levels (Schwarz et al., 2011; Jablonsky et al., 2016). However, none of these organisms are ecologically relevant, especially concerning the marine environment, which is affected by universal but also specific environmental parameters as compared to terrestrial and freshwater ecosystems.

In oceanic waters, the cyanobacterial community is largely dominated by two genera, *Prochlorococcus* and *Synechococcus*, which are only distantly related to their model freshwater counterparts. While *Prochlorococcus* mainly thrives in warm, nutrient-poor oceanic waters, *Synechococcus* can colonize a broader range of ecological niches, extending from the equator to subpolar waters as well as from estuaries to oligotrophic waters of the ocean (Flombaum et al., 2013; Xia et al., 2015; Paulsen et al., 2016). This abundance, ubiquity and the availability of many genomes and strains in culture make *Synechococcus* one of the most relevant model microorganisms to study the response to variations of environmental conditions in the marine ecosystem. Quite a few transcriptomic studies have been conducted on *Prochlorococcus* and *Synechococcus* strains (Tolonen et al., 2006; Lindell et al., 2007; Zinser et al., 2009; Tetu et al., 2009, 2013; Blot et al., 2011; Thompson et al., 2011; Mella-Flores et al., 2012; Reistetter et al., 2013; Voigt et al., 2014; Stazic et al., 2016; Lambrecht et al., 2019), but they have mainly focused on the effect of single environmental factors. Here, the well-characterized marine *Synechococcus* strain WH7803, a warm temperature-adapted ecotype, which also has the advantage of being axenic (Kana and Glibert, 1987a, b; Kana et al., 1988; Garczarek et al., 2008; Blot et al., 2011; Mella-Flores et al., 2012; Pittera et al., 2018), was selected to study the effect of various stress conditions, high light (HL), UV, low (LT) and high (HT) temperatures, on cultures previously acclimated to either low light (LL) or HL conditions, as well as to assess the effect of diel variations, as triggered by a modulated light/dark (L/D) cycle. These parameters are indeed well known to affect the physiology of this organism but the regulatory processes involved remain poorly studied. A comparison of global gene expression profiles allowed us to identify the common and specific transcriptomic responses to the different conditions. This study notably revealed that cells previously acclimated to HL seem to be better prepared than LL-acclimated cells to sustain an additional light stress but not a LT stress, which in contrast seems to induce a synergic effect with the HL treatment, as previously observed for oxidative stress (Blot et al., 2011).

## Materials and Methods

### Culture Conditions

*Synechococcus* sp. WH7803, an axenic strain retrieved from the Roscoff culture collection (RCC752)^1^, was grown in PCR-S11 culture medium (Rippka et al., 2000) supplemented with 1 mM sodium nitrate and 1 μg L^–1^ vitamin B12. Cultures were acclimated for at least 2 weeks at 22°C and 20 μmol photon m^–2^ s^–1^ (hereafter LL and μE m^–2^ s^–1^) or 250 μE m^–2^ s^–1^ (hereafter HL) provided by multicolor (cool white: 6500K, blue: 470 nm, green: 530 nm) LED systems (Alpheus, France). For each growth irradiance, a 9 L exponentially growing culture was split in 500 mL aliquots into either 1 L polycarbonate flasks (Nalgene, St. Louis, MO, United States) or 1 L Erlenmeyer quartz flasks (for UV treatments only; Atelier Jean Premont, Bordeaux, France) and left under their initial growth irradiance overnight before being submitted to light, UV or temperature stress conditions and sampled at different times (Supplementary Figure S1 and Supplementary Table S1) for various parameters (see below). UVA and UVB radiations, generated from UVA-351 and UVB-313 fluorescent bulbs (Q-Panel Lab products, Cleveland, OH, United States), were measured between 280–320 and 320–400 nm, respectively using a USB2000 spectroradiometer (Ocean Optics, EW Duiven, Netherlands). In order to estimate the cell recovery capacities, HL and UV stressed cultures were then shifted back to their initial growth condition and sampled after 1h (R1) and 24h (R24) of recovery. Low (13°C, hereafter LT) and high temperature (30°C, hereafter HT) stresses were performed in temperature-controlled chambers (Liebherr-Hausgeräte, Lienz, Austria). All of these experiments were conducted in triplicate.

For diel cycle experiments, run at two temperatures (21 and 27°C), a bell-shaped 12/12 h L/D cycle, triggering a proper synchronization of cell division, was generated using the multicolor LED systems and the Ether software (Alpheus, France). The maximal irradiance (at virtual noon) was set at 669 μE m^–2^ s^–1^. Two replicate cultures per temperature were acclimated to L/D cycles for at least 2 weeks prior to starting monitoring the different parameters. During the experiment, cultures were diluted with a continuous input of fresh medium, in order to maintain cells in exponential growth throughout the whole sampling period. In order to study the kinetics of the response of cells to light fluctuations, cultures were sampled at virtual 6:00, 9:00, 12:00, 15:00, 18:00, 20:00, 22:00, and 2:00 over 4–5 days for measuring a variety of parameters described below and samples from two out of these days were selected for transcriptomic analyses (see Table 1). Additionally, for flow cytometric analyses, 200 μL samples were transferred every hour using an Omnicoll Fraction Collector (Lambda, Brno, Czechia) into microtubes maintained at 4°C by Peltier effect and containing 0.25% glutaraldehyde grade II (Sigma Aldrich, St Louis, MO, United States), then stored at −80°C until analysis.

**TABLE 1:** Description of the samples used for transcriptomic analyses.

Acclimation condition	Stress condition	Sampling times (hours)	Number of replicates
LL	HL	0, 0.3, 1, 3, 6	3
LL	LLUV	0, 0.3, 1, 3, 6	3
LL	LLLT	0, 12, 24, 48, 72	3
LL	LLHT	0, 12, 24, 48, 72	3
HL	HLUV	0, 0.3, 1, 3, 6	3
HL	HLLT	0, 4, 8, 16, 24	3
HL	HLHT	0, 4, 8, 16, 24	3
L/D 21°C	n.a.	6, 9, 12, 15, 18, 20, 22, 2	4
L/D 27°C	n.a.	6, 9, 12, 15, 18, 20, 22, 2	4

*For shift experiments, triplicate cultures were acclimated to medium temperature (22°C) and either low light (LL, 20 μE m^–2^ s^–1^) or high light (HL, 250 μE m^–2^ s^–1^) conditions and submitted to low temperature (LT, 13°C), high temperature (HT, 30°C), ultraviolet radiations (UV-A, 3 W m^–2^; UV-B, 0.3 W m^–2^ as well as HL for the LL-acclimated culture only; Supplementary Figure S1). Expression values measured on cultures harvested at T0 were used as references to measure differential gene expression at different time points (Supplementary Table S2). For 12 h/12 h L/D cycle experiments, sampling times correspond to the time of the day, with light period starting at 6:00 and dark period at 18:00 and the four biological replicates consist in two replicate cultures and two different days for each temperature condition (Supplementary Table S1). Differential gene expression at different time points was calculated using as references either the 6:00 time point for each temperature condition or the corresponding time at 21°C when comparing the two temperature conditions (Supplementary Table S2).*

### Flow Cytometry

*Synechococcus* cell concentration was measured by flow cytometry as previously described (Marie et al., 1999) using a FACSCanto II flow cytometer (Becton Dickinson, San Jose, CA, United States) with a laser emission set at 488 nm and distilled water as sheath fluid (Supplementary Table S1).

### *In vivo* Fluorescence Measurement

PSII quantum yield (F_V_/F_M_) was measured for all samples (Supplementary Table S1) upon excitation at 520 nm with a Pulse Amplitude Modulation fluorometer (Phyto-PAM I, Walz, Effeltrich, Germany) equipped with a chart recorder (Vernier, LabPro, Beaverton, OR, United States), as previously described (Mella-Flores et al., 2012). Briefly, after 3 min acclimation in the dark, cultures were exposed to modulated light and basal level fluorescence (F_0_) was then measured. The maximal fluorescence levels (F_M_) were measured after adding 100 μM of PSII blocker 3-(3,4-dichlorophenyl)-1,1-dimethylurea (DCMU) and by triggering saturating light pulses (655 nm; 2000 μE m^–2^ s^–1^). The PSII quantum yield was calculated as:

F/VF=M(F-MF)0/FM

Fluorescence emission spectra were recorded for all samples (Supplementary Table S1) between 545 and 750 nm upon excitation at 530 nm (λ_max_ absorption of phycoerythrobilin) with an LS-50B spectrofluorometer (Perkin-Elmer, Waltham, MA, United States) equipped with a red sensitive photomultiplier as previously described (Six et al., 2004). In order to determine the coupling of phycobiliproteins and to observe any dismantlement of phycobilisome (PBS) rods, fluorescence emission spectra were then used to calculate the phycoerythrin (PE, λ_max_ = 565–575 nm) to phycocyanin (PC, λ_max_ = 645–655 nm) ratio as well as the PC to PBS terminal acceptor (TA; 680 nm). The latter ratio reflects the energy transfer from phycocyanin to reaction center chlorophylls (Pittera et al., 2017).

### PSII Repair Rate Measurements

PSII repair rate was calculated for all samples (Supplementary Table S1) but in two different ways, depending on stress duration. For short-term stresses (HL and UV stress, 6-h stress experiments), PSII repair rate was estimated over the 6 h of stress. Two aliquots of 20 mL were sampled at T0. While the first one was used as a control, lincomycin (protein synthesis inhibitor) was added to the second one to a final concentration of 0.5 mg mL^–1^ and the F_V_/F_M_ parameter was monitored at each timepoint. The difference between the coefficients of the exponential curves fitted on F_V_/F_M_ measurements was used as proxy of the PSII repair rate. For the long-term (1 to 4-days) experiments (LT and HT stress) and for L/D cycles, six aliquots of 2 mL were taken at each timepoint, placed in 8-wells plates and lincomycin was then added to three of them, as described above. All aliquots were placed in the same conditions as the rest of the culture and F_V_/F_M_ was monitored after 15, 30 min, and 1 h, respectively.

### Pigment and Lipid Analyses

Pigment content was measured by HPLC for all treatments except the L/D cycles (Supplementary Table S1) using the protocol described in Pittera et al. (2018). Furthermore, previously published lipid data, corresponding to the LLLT and LLHT treatments (see Supplementary Figures S3,S4 in Pittera et al., 2018) were also used for correlation analyses (see below).

### RNA Extraction

RNA extractions were performed from 150 mL of culture harvested by centrifugation in an Eppendorf 5804R (7 min, 10,414 × g, 4°C) then in an Eppendorf 5417R centrifuge (2 min, 20,817 × g, 4°C). The cell pellet was then resuspended in 1 mL Qiazol (Qiagen, Valencia, CA, United States) and quickly frozen in liquid nitrogen. The total duration of cell harvesting was kept below 15 min. After thawing the tubes at 65°C, followed by incubation and vortexing for 5 min at this temperature, total RNA was extracted using the Direct-Zol kit (Zymo Research Corp, Proteigene) as recommended by the manufacturer. Three successive DNase treatments were performed on the Zymo-Spin^TM^-IIC Column using the Qiagen RNase-free DNase Set (Qiagen), followed by elution from the column in 30 μL DEPC-treated water. RNA quantification and quality check were performed using a NanoDrop^®^ ND-1000 (Thermo Fisher Scientific) and a BioAnalyzer 2100 with the RNA 6000 Nano Kit (Agilent, Santa Clara, CA, United States), respectively. RNA samples were then frozen in liquid nitrogen and stored at −80°C.

### Library Preparation and Sequencing

Ribodepletion was performed at the Genotoul platform (Toulouse, France) from 5 μg of total RNA using the Ribo-Zero Bacteria Magnetic Kits (Illumina) in the presence of RiboGuard RNase Inhibitor (Epicentre) and treated RNA samples were purified and concentrated using RNA Clean & Concentrator^TM^-5 columns (Zymo Research). Ribo-depleted RNA was then quantified by Qubit (RNA HS Assay Kit, Thermo Fisher Scientific) and quality check was performed using a Bioanalyzer (RNA Pico kit) or a Labchip GX. Libraries were then prepared from 100 to 400 ng of RNA using the Illumina TruSeq Stranded mRNA kit and sequenced as 150 bp paired-end reads on Illumina HiSeq 3000 System (Illumina, San Diego, CA, United States) at Genotoul (Toulouse, France). 18.5 ± 4.7 × 10^6^ paired-end reads were generated per sample.

### RNA-Seq Analysis

Reads quality check was performed with the FastQC tool (Andrews, 2015). Reads smaller than 50 nt and with a mean quality score lower than 25 were excluded using Prinseq v0.20.4 (Schmieder and Edwards, 2011). Illumina adapters, constituting 23.5 ± 4.5% of reads, and rRNAs, constituting 5.0 ± 5.9% of all reads, were removed with Cutadapt v1.8.3 (Martin, 2011) and SortMeRNA v2.1 (Kopylova et al., 2012), respectively. The remaining reads were then mapped to the reference genome of *Synechococcus* sp. WH7803 using Bowtie2 v2.2.9 software (Langmead and Salzberg, 2012) with ‘–non-deterministic –end-to-end –sensitive’ parameters and by selecting only the properly paired reads (i.e., SAM flagged as 99, 147, 163, or 83). Read count tables were obtained using HTSeq Count v0.6.0 (Anders et al., 2015) with following parameters: ‘–stranded = reverse -a 10 -m intersection-nonempty’. Gene expression levels between stress and control conditions as well as adjusted p-values based on the Benjamini–Hochberg procedure (Benjamini and Hochberg, 1995) were determined using SARTools v1.4.0 (Varet et al., 2016) with embedded DESeq2 v1.14.1 (Love et al., 2014). Genes were considered as differentially expressed (DE) if the adjusted *p*-value was ≤0.05 and the absolute value of log_2_ fold change (log_2_FC) was ≥1. The RNA-seq dataset is available as raw and processed data in the SRA and GEO databases, respectively (see section Data Availability Statement) and can be visualized using JBrowse (Buels et al., 2016) in the Cyanorak v2.1 database^2^.

### Co-expression Network Model Analysis

Weighted gene correlation network analysis (WGCNA; Langfelder and Horvath, 2008) was performed on shift experiments to define gene subnetworks, called modules, based on gene expression patterns. A signed adjacency matrix between genes was calculated and Pearson correlations were weighted by taking their absolute value and raising them to the power β = 12 to optimize the scale-free topology network fit. In order to identify groups of genes, whose expression was correlated to the biological, physical or chemical traits available for this experiment (including previously published lipid data; Pittera et al., 2018), the pairwise Pearson correlation coefficients between the principal component of each module, referred to as the module *eigenvalue* (ME), and these traits was then calculated using the R package WGCNA (Langfelder and Horvath, 2008). Modules were then filtered using a partial least square (PLS) regression, a dimensionality-reduction method that aims at determining predictor combinations with maximum covariance with the response variable. The predictors were ranked according to their value importance in projection (VIP), as previously described (Guidi et al., 2016). Furthermore, the UpSet R package was used to count and plot the number of genes per module that were DE in a given set of conditions (e.g., LLHT and HLLT) by considering only genes significantly DE in at least one time point of each condition for this set of conditions.

### Relationship Between Genomic Features and Transcriptomic Responses to Different Stresses

Transcriptomic data were synthesized as a circos plot integrating: (i) core genes, as determined based on the comparison of the 81 non-redundant genomes of the Cyanorak v2.1 database^2^ (Doré et al., under revision), (ii) WGCNA module and submodule membership, (iii) predicted operons as extracted from ProOpDB (Taboada et al., 2012), (iv) ‘cyanorons’ that we defined as suites of ≥4 adjacent genes on the genome that belong to the same WGCNA module and displayed at least half of all time points for a given stress with a similar expression pattern (e.g., all 4 adjacent genes belonging to module Blue and all displaying either a Log_2_FC > 1 or < 1).

## Results

### Photophysiological Response of *Synechococcus* sp. WH7803 to Variations in Light and Temperature Conditions

**FIGURE 1:**
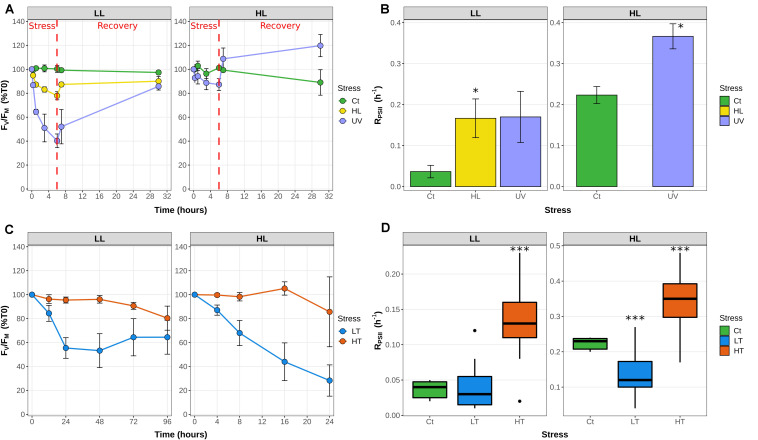
Variation of the photophysiological parameters in response to changes in light or thermal conditions in *Synechococcus* sp. WH7803 acclimated to low or high light. **(A,C)** PSII maximal photochemical yield (F_V_/F_M_) expressed as % of initial F_V_/F_M_ after a shift **(A)** to high light or UV radiations and **(C)** to low temperature (LT: shift from 22°C to 13°C) or high temperature (HT; shift from 22 to 30°C). The dashed line indicates the time (6 h) at which cultures submitted to light stresses were shifted back to their initial light conditions for recovery. **(B,D)** Cumulative photosystem II repair rate (RPSII), (B) during the 6 h of light stress and (D) during 1 h for low and high temperature shifts. While for panels **(A–C)**, data represent averages and standard deviations of three biological replicates, for **(D)**, boxplots represent distribution of all three independent measurements, with their medians and interquartiles. Asterisks in **(B,D)** indicate significantly different values between treatment and control conditions (Student’s *t*-test) with a single asterisk indicating *p* < 0.05, double asterisks indicating *p* < 0.01 and triple asterisks indicating *p* < 0.001. The top gray banners indicate the culture acclimation condition. These data are based on at least three independent experiments. Ct, control; LL, low light; HL, high light; UV, ultraviolet; LT, low temperature; HT, high temperature.

Cultures pre-acclimated under LL (20 μE m^–2^ s^–1^) or HL (250 μE m^–2^ s^–1^) and 22°C were subjected to a series of light or temperature shifts, as detailed in Supplementary Figure S1 and Supplementary Table S1. The quantum yield of the PSII reaction center (F_V_/F_M_), used as a proxy for PSII activity, showed that UV stress induced a much stronger response than HL stress in LL-acclimated cultures of *Synechococcus* sp. WH7803, with a decrease of the F_V_/F_M_ after 6 h of stress of 60% and 20%, respectively (Figure 1A). This F_V_/F_M_ drop was much less pronounced in HL-acclimated culture under UV stress (20%), potentially related to the much faster D1 repair rate measured in control HL- than LL-acclimated cultures (sixfold, Figure 1B). D1 repair rates were also further enhanced by UV and HL treatments in LL-acclimated conditions and by UV in HL-acclimated cells. In all cases, cultures were capable of recovering most of their PSII activity after 24 h, showing that none of these stresses were lethal for the cultures (Figure 1A). As concerns thermal stresses, HT shift, induced by an 8°C temperature increase, only had a small effect on PSII activity for cells previously acclimated to both light conditions (Figure 1C), except at the end of the LL HT experiment when cultures reached the stationary growth phase (data not shown), and these HT conditions led to a fairly high induction of the D1 repair rate compared to the control at T0 (Figure 1D). In contrast, a shift to LT, corresponding to a 9°C temperature decrease, induced a strong PSII photoinactivation with a drop in F_V_/F_M_ of 45% followed by a stabilization for LL-acclimated cells after 1 day and a continuous decrease reaching about 70% within a day for HL-acclimated cells (Figure 1C), and none of these conditions led to a significant change of the D1 repair rate of the cells (Figure 1D). Thus, in contrast to light stresses, cells previously acclimated to HL do not seem to be better prepared for thermal stress than LL-acclimated cells.

Photoprotective and/or antioxidant pigments only showed low variations in response to light and temperature shifts. The ratios of the β-carotene (β-car), zeaxanthin (zea) and β-cryptoxanthin (β-crypto) to chlorophyll (chl) *a* only showed low variations in response to light and temperature shifts, with (i) a slight increase of the two latter ratios and decrease of the first one in response to HL in LL-acclimated cultures, (ii) an increase of the sole β-crypto/chl *a* ratio in response to UV and (iii) a decrease of β-car/chl *a* in response to LT in both light acclimated cultures (Supplementary Figure S2). Furthermore, variations of the ratios of the fluorescence emission peaks were used to assess the efficiency of the energy transfer within the PBS rods (PE/PC) as well as between the basal part of the rods and the PSII reaction center (PC/TA), as measured by fluorescence leaks between these phycobiliprotein subunits (Pittera et al., 2017; Supplementary Figure S3). While HL and UV had a rapid and stronger effect on the base of the PBS rods and/or on the connection of the PBS to the thylakoid membrane (PC/TA) than at the rod level (PE/PC, Supplementary Figures S3A,B), LT seemingly induced a progressive disruption of the PBS, both at the rod level and at the core level (Supplementary Figures S3C,D). In contrast, the shift to HT had virtually no effect on the PBS structure.

### Comparison of the Photophysiological Response of *Synechococcus* sp. WH7803 to a L/D Cycle at Two Temperatures

**FIGURE 2:**
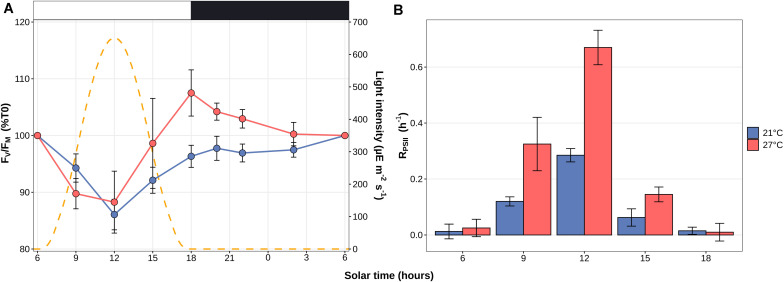
Daily variations of the photosystem II maximal quantum yield (F_V_/F_M_) and D1 repair rates for *Synechococcus* sp. WH7803 cells acclimated to a modulated 12 h/12 h L/D cycle at 21 or 27°C. **(A)** PSII maximal photochemical yield (F_V_/F_M_) expressed as % of F_V_/F_M_ at 6 a.m. **(B)** Photosystem II repair rate (RPSII). Note that this parameter was not measured during the night since a previous study showed that there is no repair during this period (Mella-Flores et al., 2012). Light and dark periods (μE m^–2^ s^–1^) are indicated by white and gray background on the figure and by a dashed line during the light period showing the modulation of light intensity. Error bars indicate standard deviation for four biological replicates.

At both temperatures, the F_V_/F_M_ ratio showed a cyclic evolution, reaching a minimum value during the light period. However, while at 21°C the F_V_/F_M_ mirrored the light curve with a minimum at noon and fairly constant higher values during the whole dark period, at 27°C F_V_/F_M_ was already close to the minimal value at 9 a.m., reached a maximum at the light/dark transition and tended to decrease during the night (Figure 2). Consistently with the LT and HT stress experiments in continuous light conditions (Figure 1D), the D1 repair rate was much higher at 27°C than at 21°C at all time-points during the light period, reaching a comparable value at 9 a.m. for the 27°C treatment to the one measured at noon for the 21°C treatment.

### Global Description of Transcriptional Responses

**FIGURE 3:**
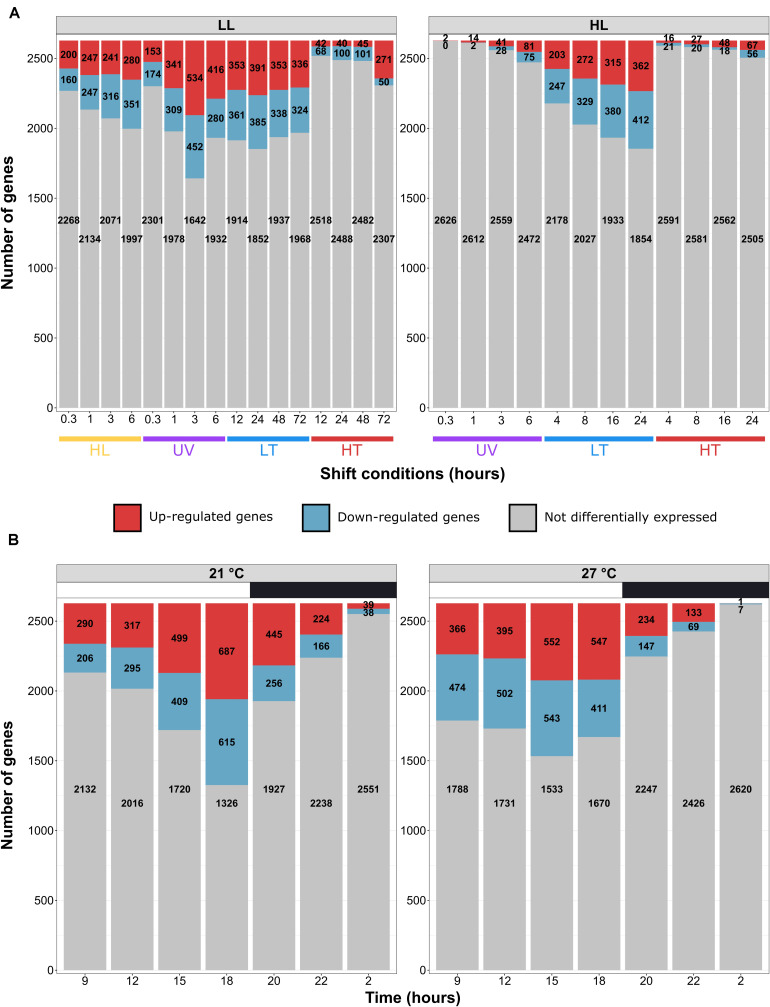
Number of differentially expressed (DE) genes in *Synechococcus* sp. WH7803 in the different tested conditions. For each condition, the number of induced, repressed and non-DE genes among the 2,634 genes of the *Synechococcus* sp. WH7803, are shown in red, blue and gray, respectively. **(A)** Shift experiments. The top gray banners indicate the prior acclimation condition of the culture: LL, low light; HL, high light. Data are based on three biological replicates. For each stress condition, numbers in x-axis correspond to ‘sampling time’ in hours and the colored bars to the ‘shift condition’ (UV, ultraviolet; LT, low temperature; HT, high temperature). **(B)** Light/dark cycle at 21 and 27°C. The top gray banners above graphs indicate the temperature used for the experiment, while the white and black banners indicate light and dark periods. Data are based on four biological replicates and differential expression was calculated relatively to the 6 a.m. data point.

Transcriptome analyses showed that 97.6 ± 1.2% of the reads were mapped to the reference WH7803 genome, resulting in average in 98.9% of the genome, including intergenic and gene coding regions, being covered by more than two reads in the different experiments, and 99.99% when considering all experiments altogether. Global analyses showed a transcriptional response proportional to the stress intensity as assessed from photophysiological measurements, i.e., there were more DE genes at the end of the stress than at the beginning (Figure 3). While light stresses (HL and UV) led to a high number of DE genes in LL-acclimated cultures (24 and 26 % of all genes at the end of stress, respectively), only a few genes were DE after UV stress in HL-acclimated cultures. Differences between LL- and HL-acclimated cultures were less conspicuous during thermal stress, both responding strongly to LT stress (25 and 29% of all genes were DE at the end of stress, respectively) and only weakly to HT stress (up to 12% and 4% DE genes, respectively). Thus, consistently with PSII activity (Figures 1A,C), the transcriptomic response showed that LL-acclimated cells were much more sensitive to UV stress than HL-acclimated cells, while LT but not HT shift seemed to constitute a very stressful condition whatever the initial light acclimation condition used.

As concerns the L/D cycle, there were large daily variations of the number of DE genes compared to the 6 a.m. time point, the highest number being reached at the light-dark transition at 21°C, while variations were less pronounced during the day at 27°C with a peak of DE genes at 3 p.m. (Figure 3B). The direct comparison between 21 and 27°C at each time point showed that differences in number of DE genes between these treatments mainly occurred during the dark period and at the L/D and D/L transitions (Supplementary Figure S4).

### Correlation Between Gene Expression and Biophysical Parameters

**FIGURE 4:**
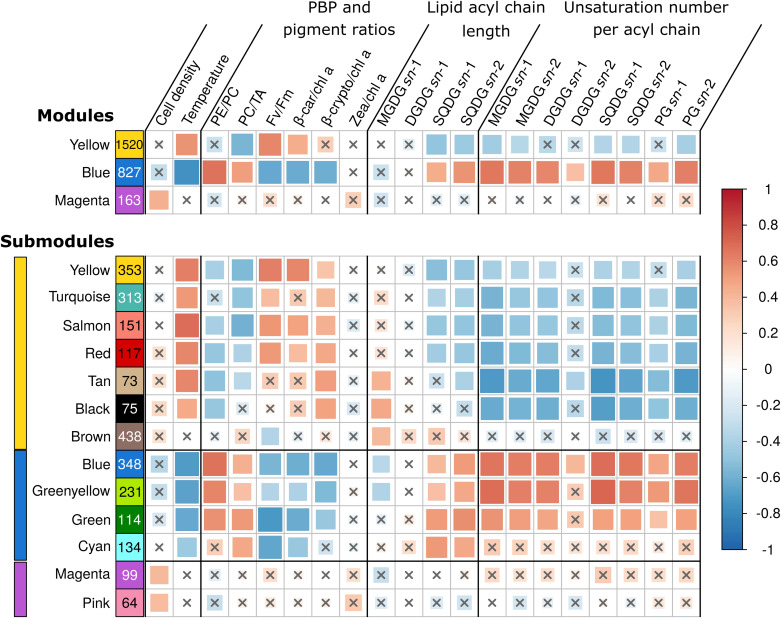
Correlations between WGCNA modules *eigengenes* and phenotypic traits. Each row represents either a gene module (top four rows) or submodules (bottom 14 rows) and number of genes in the module or submodule are specified in colored rectangles. Colored bars on left hand side indicate the corresponding module. Biophysical parameters are expressed as a percentage of the T0 of each experiment. The color scale reflects the Pearson correlation coefficient (*R*^2^). The size of the correlation squares is inversely related to the *p*-value. Genes that could not be clustered into one of the modules were assigned to the gray module (not shown), and non-significant correlations (Student adjusted *p*-value > 10^–3^) are indicated by a cross. PBP, phycobiliprotein; PC, phycocyanin; PE, phycoerythrin; TA, terminal acceptor; F_*V*_/F_*M*_, PSII maximal photochemical yield; β-car, β-carotene; chl, chlorophyll; β-crypto, β-cryptoxanthin; zea, zeaxanthin; MGDG, monogalactosyldiacylglycerol; DGDG, digalactosyldiacylglycerol; SQDG, sulfoquinovosyldiacylglycerol; PG, phosphatidylglycerol.

In order to explore transcriptome-wide responses of WH7803 in all shift conditions, we used a WGCNA approach (Langfelder and Horvath, 2008; Guidi et al., 2016). This analysis delimited three modules and 13 submodules of co-expressed genes within the global co-expression network. Each (sub)module thus contains a subset of DE genes, whose pairwise expression was highly correlated, i.e., expression of genes gathered within a given (sub)module had a high probability of varying similarly over all stress experiments (Figure 4). To further reduce the complexity of these transcriptomic data, we first focused on (i) genes with VIP scores values higher than 1 (hereafter VIP genes), i.e., the minimum set of genes of a module to obtain an association (correlation or anti-correlation) with a given trait similar to that of all genes of the module (Supplementary Table S2), on (ii) genes in each module that were significantly up or down-regulated in specific sets of conditions (hereafter called UpSet genes; Supplementary Figure S5). This approach also allowed us to determine the metabolic pathways of WH7803, based on KEGG^3^, which were most affected in response to environmental changes, as detailed in the discussion part.

**FIGURE 5:**
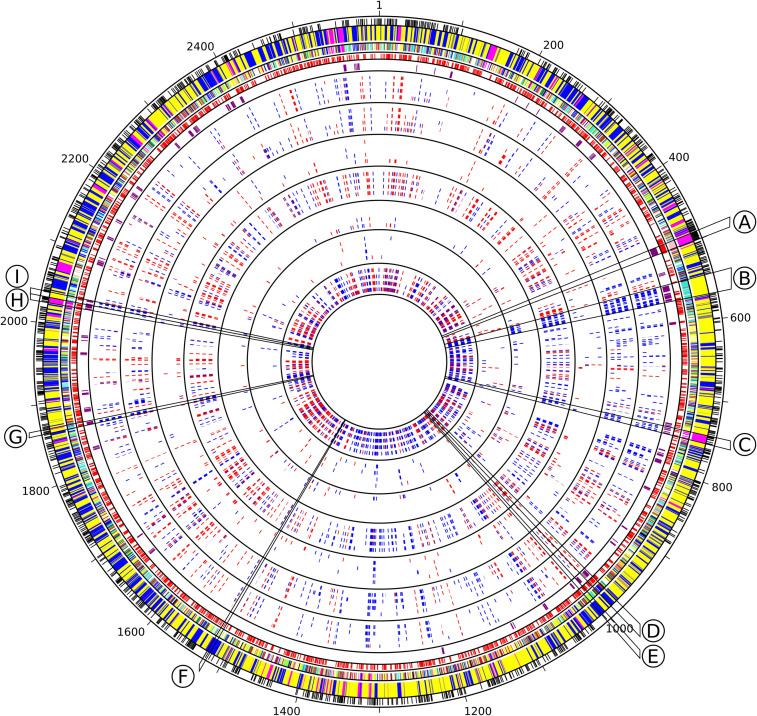
Circos representation of *Synechococcus* sp. WH7803 genomic features and transcriptomes in response to different stresses. Circles from outside to inside, separated by black lines, include core genes (black), WGCNA module and submodule membership (cf. Figure 4 for the corresponding colors), predicted operon using ProOpDB (red), Cyanorons (purple) and heatmap for differential gene expression [Log2(FC)] in all tested stress conditions displayed in the following order: LLHL (0.3, 1, 3, 6), LLUV (0.3, 1, 3, 6), LLHT (12, 24, 48, 72), LLLT (12, 24, 48, 72), HLUV (0.3, 1, 3, 6), HLHT (4, 8, 16, 24), HLLT (4, 8, 16, 24), with number between brackets indicating ‘sampling time’ in hours. Slices delineated by black lines indicate group of genes mentioned in the text involved in specific functions: **(A)** ribosomal proteins, **(B)** phycobilisome rod biosynthesis, **(C)** CO_2_ fixation (carboxysomes and RuBisCO), **(D)** multiprotein Na^+^ antiporter subunits, **(E)** ABC-type Mn^2+^/Zn^2+^ transport system, **(F)** exodeoxyribonuclease V (RecBCD complex), **(G)** cytochrome *c* oxidase subunits, **(H)** ATP synthase, **(I)** Allophycocyanin. Graduations correspond to the position of the genes on the genome.

The yellow module was correlated to the β-car/chl *a* ratios, F_V_/F_M_, temperature and anti-correlated to the PC/TA ratios, to the glycolipid sulfoquinovosyldiacylglycerol (SQDG) *sn*-1 and *sn*-2 acyl chains lengths, as well as to the number of unsaturations on both *sn*-2 and/or *sn*-1 acyl chains of SQDG and most other glycolipids (Supplementary Table S2 and Figure 4). Among the 1,520 genes of this module, 102 were VIP and were dominantly photosynthetic genes notably involved in the synthesis of pigments, PSI and PSII, cytochrome *b*_6_/*f*, plastocyanin as well as PBS subunits and linkers, most of them being downregulated and belonging to the turquoise submodule. Of note, most genes coding for PBS components were gathered into two ‘cyanorons’, i.e., were co-expressed and adjacent on the genome (Figure 5, sections B and H). The fact that many genes involved in the light-dependent reactions of photosynthesis were downregulated in HL, UV, and LT treatments suggests that these stresses all had a strong inhibitory effect on this central metabolic process. Notable exceptions, all clustered in the brown submodule, were in contrast upregulated in response to most treatments (Supplementary Table S2). These include genes involved in the turnover of the PSII reaction center (a D1:2 encoding *psbA* gene, a *psbD* gene copy and the D1 repair gene *ftsH2*), but also three genes coding for high-light-inducible proteins (HLIPs) as well as *ubiH* involved in plastoquinone biosynthesis. VIP genes of this submodule also included several chaperones and proteases (GroL1, GroL2, ClpB1, ClpC, DnaK3 and a small heat shock protein, HSP20) as well as three genes coding for sigma factors (*rpoD4, 6 and 7*), all three latter genes also showing a notable diel expression (Supplementary Table S2).

When looking at the 1,039 UpSet genes of this module, one can see that 149 were induced (brown submodule, 110 genes) or repressed (turquoise submodule, 39 genes) specifically in LLUV and 60 more were mostly induced in LLUV and LLHT, the latter gene set being upregulated mainly at the last data point of the high temperature stress, corresponding to cells in stationary phase (Supplementary Figure S5). These categories notably included genes coding for five ATP-dependent Clp proteases (ClpP1-4, ClpS), the stress-inducible DNA-binding protein DpsA, as well as seven out the 20 subunits of the NADH dehydrogenase I (Supplementary Table S2). Furthermore 42 UpSet genes were DE in all stress conditions except HT treatments, while 84 others were DE in all but HT and HLUV, the latter conditions previously shown to be the least stressful conditions in this experimental setup (Figure 3A). Both sets of genes were mainly downregulated and essentially encompassed genes involved in the abovementioned photosynthetic light reactions, but also a few genes involved in heme/vitamin biosynthesis (five genes), cell division (four genes) as well as the universal stress family protein UspG.

**FIGURE 6:**
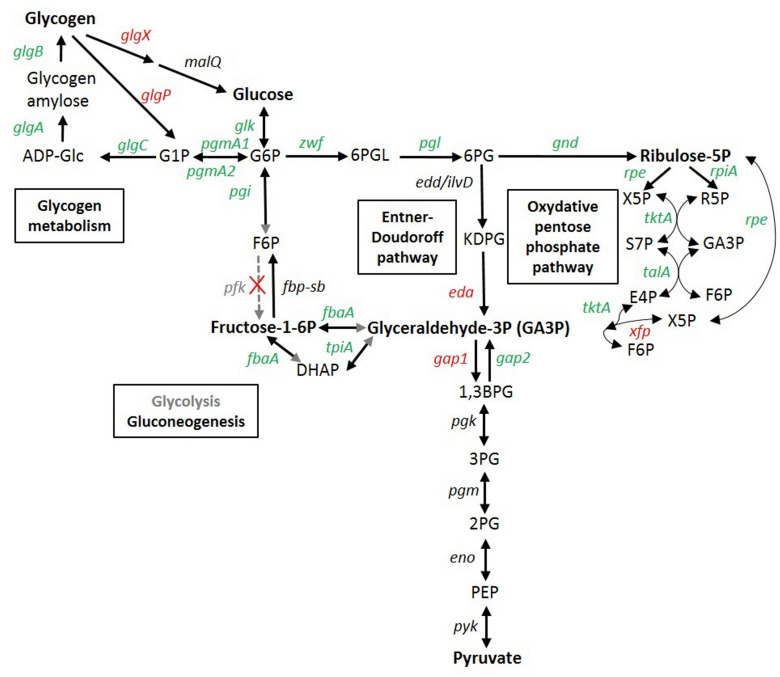
Transcriptomic response of glycogen and glucose metabolism genes in *Synechococcus* sp. WH7803. In red, genes induced in at least one stress condition (usually LLLT); in green, genes repressed in at least one stress condition; in black, genes not significantly differentially expressed whatever the condition; in gray, no obvious candidate was found in the genome for this function, so the reactions shown by gray arrows or arrowheads likely do not occur in this strain. Gene products are the following: *glgA*, glycogen synthase; *glgB*, 1,4-alpha-glucan branching enzyme; *glgC* glucose-1-phosphate adenylyltransferase; *glgP*, glycogen phosphorylase; *glgX*, glycogen isoamylase; *malQ*, 4-alpha-glucanotransferase; *pgmA*, phosphoglucomutase; *glk*, glucokinase; *pgi*, glucose-6-phosphate isomerase; *fbp*, fructose-1,6-bisphosphatase; *pfk*, phosphofructokinase; *fbaA (a.k.a. cbbA)*, fructose-bisphosphate aldolase; *tpiA*, glycogen isoamylase; *zwf*, glucose-6- phosphate dehydrogenase; *pgl* (a.k.a *devB*) 6-phosphogluconolactonase; *edd/ilvD* (potential bifunctional 6-phosphogluconate dehydratase/dihydroxy-acid dehydratase); *eda*, 2-keto-3-deoxygluconate-6-phosphate aldolase; *gnd*, 6-phosphogluconate dehydrogenase; *rpiA*, ribose-5-phosphate isomerase; *rpe*, pentose-5-phosphate-3-epimerase; *talA*, transaldolase; *tktA*, transketolase, *xfp*: xylulose 5-phosphate/fructose 6-phosphate phosphoketolase; *gap1*, glyceraldehyde-3-phosphate dehydrogenase (catabolic reaction) *gap2*, glyceraldehyde-3-phosphate dehydrogenase (anabolic reaction); *pgk* phosphoglycerate kinase; pgm (=*gpmB)*, phosphoglycerate mutase; *eno*, enolase; *pyk*, pyruvate kinase. ADP-Glc, ADP-glucose; G1P, glucose-1-phosphate; G6P, glucose-6- phosphate; F6P, fructose-6-phosphate; DHAP, Dihydroxyacétone phosphate; GA3P, glyceraldehyde-3-phosphate; 6PGL. 6-phosphogluconolactone; 6PG, 6-phosphogluconate; KDPG, 2-keto-3-deoxygluconate-6-phosphate aldolase; X5P, xylulose-5-phosphate; R5P, ribose-5P; S7P, sedoheptulose-7-phosphate; E4P, erythrose-4-phosphate; 1,3 BPG, 1,3, bisphosphoglycerate; 3PG, 3-phosphoglycerate; 2PG, 2-phosphoglycerate; PEP, phosphoenolpyruvate. Adapted from Osanai et al. (2007), Chen et al. (2016), and the KEGG database (www.genome.jp/kegg/pathway.html).

The blue module, gathering 827 genes, exhibited a mirrored correlation pattern compared to the yellow one, except that a significant correlation was found for several parameters, which were anticorrelated with the yellow module but not significantly (Figure 4). Although the WGCNA analysis resulted in four submodules within the blue module, their analysis did not reveal any clear trend with regard to the different treatments. Among the 135 VIP genes of this module, most of them were upregulated and numerous genes were involved in central metabolism, including DNA replication and repair, protein fate, oxidative stress response, fatty acid biosynthesis as well as sugar catabolism (Figure 6). Also notable in this module were several two-component systems, three genes of the SUF (sulfur utilization factor) system (Banerjee et al., 2017), as well as several members of the DNA/RNA helicase family, which by rearranging the secondary structure of nucleic acids, could play a role in the response to variations of environmental conditions (see e.g., Chamot et al., 1999; Chamot and Owttrim, 2000; Owttrim, 2006). This includes CrhR, an ortholog of Slr0083, known to be upregulated by cold stress in *Synechocystis* sp. PCC 6803 (Georg et al., 2019), which was indeed also the case for HL-acclimated WH7803 cells.

Consistently with the fact that the blue module was strongly anticorrelated to temperature, UpSet genes of this module gathered 55 genes seemingly specific to LT stresses (LLLT and HLLT), while 41 more genes were found only in LLLT and 49 only in HLLT conditions (Supplementary Figure S5 and Supplementary Table S2). Genes among these categories were mostly upregulated and notably included *ctpA*, encoding the carboxyl-terminal processing protease involved in the maturation of the PSII core subunit D1, the lycopene β-cyclase encoding gene *crtL-b*, several genes involved in DNA replication recombination and repair, numerous glycosyltransferases, potentially involved in cell wall biosynthesis as well as genes involved in biosynthesis and regulation of vitamins, including biotin (B7; Rodionov et al., 2002) and pseudocobalamin (B12; Helliwell et al., 2016). Also notable were several transporters constituting ‘cyanorons’ on the circos representation : an ABC-type Mn^2+^/Zn^2+^ transporter (Figure 5, section E) as well as several subunits of the multiprotein Na^+^/H^+^ antiporter (*mrpCDF*, Figure 5, section D), the latter genes being mostly downregulated during the day and upregulated during the night. Interestingly, the blue module also encompassed 49 genes differentially regulated specifically in LL-acclimated cultures submitted to UV stress. While half of them were downregulated, including *ftsZ* involved in ring formation during cell division, the other half were upregulated and included a few photosynthetic genes and several ABC transporters (Supplementary Table S2).

The magenta module, which encompasses 163 genes, including 12 VIP, was significantly correlated with only one parameter, namely the variation of cell density compared to T0. VIP genes notably include two genes involved in the response to oxidative stress, two coding for subunits of the DNA-directed RNA polymerase (the other two subunits being also clustered in the magenta module) as well as *rbcL*, encoding the large subunit of the RuBisCO, the latter being upregulated in response to the LLHL treatment but downregulated in LLUV, LLLT, and HLLT. Additionally, a large number of UpSet genes (24), mainly found in the pink submodule, seemed to be upregulated significantly in LLHL but non-significantly in other conditions (Supplementary Table S2). This included genes coding for 19 out of the 53 ribosomal proteins, while 19 others were also in the pink submodule, all these genes constituting the second largest ‘cyanoron’ of the WH7803 genome (Figure 5, section A). In contrast, the magenta submodule essentially gathered genes upregulated in LLHL but downregulated or not DE in most other shift conditions. It contains (i) not only the abovementioned *rbcL* gene but also genes encoding the second subunit of the RuBisCO (*rbcS*) and most carboxysome shell proteins, altogether constituting another large ‘cyanoron’ (Figure 5, section C), (ii) all genes coding for the different subunits of the ATP synthase (Figure 5, section G) as well as (iii) numerous genes involved in nitrogen uptake (*focA*, *amt1*, *nrtP*), assimilation (*cynH, S, glnA, nirA, narB, mobA*) and regulation (*ntcA*). Thus altogether, it seems that all critical pathways involved in CO_2_ fixation, ATP production and nitrogen metabolism were specifically activated in response to HL treatment (and showed a peak of expression at 9 a.m. in L/D), while being downregulated in response to other treatments.

## Discussion

### General Response of Marine *Synechococcus* to Various Stress Conditions

Understanding how marine phytoplankton will react to variations in the physico-chemical properties of the marine ecosystem, some of which being triggered by the ongoing global changes, constitutes one of the major current challenges in microbial ecology. For this reason, we investigated the response of WH7803, one of the best physiologically characterized marine *Synechococcus* isolates (Kana and Glibert, 1987a, b; Kana et al., 1988; Garczarek et al., 2008; Blot et al., 2011; Mella-Flores et al., 2012; Pittera et al., 2018) to various ecologically realistic stresses, i.e., stresses that cells could experience in the field, e.g., during mixing events, as well as to L/D cycles. Physiological responses to these conditions showed that a shift from 22°C to 30°C (LLHT and HLHT) actually did not constitute a stress for this strain, as shown by a moderate change in F_V_/F_M_ (Figure 1C) and the low number of DE genes (Figure 3A). In contrast, a shift from 22 to 13°C induced a strong stress response, whatever the initial light acclimation conditions, although the cells were seemingly able to maintain ∼60% of their F_V_/F_M_ over the time course of the experiment when they were initially acclimated to LL, while HL-acclimated cells appear to be more strongly affected, with a sharp and continuous decrease of their F_V_/F_M_. Consistent with a previous study of the oxidative stress response of WH7803 cells after a pre-acclimation at LL or HL (Blot et al., 2011), a synergic effect seems to occur between light history and low temperature stress. While this behavior was attributed to the generation of reactive oxygen species (ROS), inducing both direct damages to the reaction centers and inhibition of their repair cycles, the deleterious effect of ROS could be even amplified by the slowing down of the metabolism, and thus of the D1 turnover, at LT. In contrast, cells acclimated to HL seem to be better prepared to sustain UV stress than LL-acclimated cells since a much lower number of genes were DE at all time points of UV stress in HL- than in LL-acclimated cultures (Figure 3A) and there was a much smaller drop in F_V_/F_M_ in HL conditions, partially attributable to a sharp increase in the D1 repair capacity of the cells (Figure 1A). As concerns the L/D cycle, the F_V_/F_M_ ratio varied strongly during the day, as previously described (Mella-Flores et al., 2012). The comparison between 21 and 27°C revealed that the amplitude of variations of both F_V_/F_M_ and D1 repair rate was more pronounced at higher temperature (Figure 2). In contrast, the number of DE genes varied much more at 21°C with a peak at the L/D transition (18:00), while a large number of genes were DE during the whole light period at 27°C (Figure 3B), which tends to support the synergic effect of light and temperature observed in continuous light conditions. Analyses of the 154 transcriptomes also revealed the common and specific responses to the different tested conditions and the respective roles of various metabolic pathways (based on KEGG) either particularly represented in the WGCNA modules based on TIGR roles and Cyanorak functional categories or previously known to be involved in stress responses. These pathways are detailed in the following sections.

### Chaperones

Chaperone- and protease-encoding genes were among the most strongly induced genes in response to all stressful conditions (HL, UV, and LT) but also at the end of the exponential growth phase (LLHT-D3). Among these, the two *clpB1* gene copies as well as genes encoding the GroEL/ES system (*groS, L1, L2*), the small heat shock protein (HSP20), HtpG, three out of seven DnaJ (*dnaJ1*, *2* and *5*), one out of three DnaK (*dnaK3*) as well as the serine endoprotease *degQ*, were activated in all stressful conditions, while *clpP1*-4 and *clpC* seem to be more specific to UV. Consistent with previous studies, the expression of *clpX*, encoding a regulatory ATPase/chaperone interacting with ClpP, was repressed under LLHL, LLUV, and HLLT (Schelin et al., 2002; Blot et al., 2011).

### Photosynthesis Light Reactions

As expected from the central role of photosynthesis in the metabolism of cyanobacteria, photosynthetic genes were among the most DE in the various stresses. Most genes encoding PSI, cytochromes *b*_6_/*f* and phycobilisomes were strongly downregulated in LL-acclimated cells in response to HL, UV and LT stresses, with the notable exception of the four phycocyanin-specific phycobilin lyases *cpcS* and *rpcE-F-T* (Six et al., 2007), which were slightly upregulated by UV stress. In contrast, genes involved in PSII biosynthesis displayed a much more variable response to stress, likely due to the particular sensitivity of PSII reaction centers to photodamage (Supplementary Table S2). Indeed, two competing processes are at work in the transcriptional response of PSII genes to stress: (i) the *de novo* assembly of all PSII subunits and its cofactors and (ii) the replacement of damaged D1 proteins, so-called ‘PSII repair’, which involves partial disassembly and subsequent rebuilding of PSII (Nickelsen and Rengstl, 2013; Mabbitt et al., 2014; Supplementary Figure S6). Like for other components of the photosynthetic apparatus, the neo-biogenesis of PSII components seems to be repressed in response to all stresses, as suggested by the downregulation of *psbB-I-K-M-O-T-U-V-X-Y-Z, cyanoP-Q* as well as *psb30* and *psb32* genes. Yet, the expression pattern of all other PSII genes was seemingly influenced by the D1 repair process. This includes the three *psbA* gene copies encoding the D1:2 isoform, which were strongly induced in all treatments in contrast to the sole gene encoding the more photosynthetically efficient but less stress-resistant D1.1 isoform (Garczarek et al., 2008; Kós et al., 2008). Similarly, as previously observed for *Synechococcus* sp. PCC 7942 (Bustos and Golden, 1992) and *Synechocystis* sp. PCC 6803 (Viczián et al., 2000), the two genes encoding D2 proteins displayed a distinct DE pattern. Indeed, while the *psbD* copy in operon with *psbC* was weakly but specifically induced by UV stress, the monocistronic *psbD* gene copy was in contrast almost as strongly upregulated as the pool of D1:2-encoding *psbA* genes under LLHL, LLUV, and LLHT conditions. This suggests that D2 needs to be synthesized at a similar rate as D1:2 in order to maintain the PSII active in LL-acclimated cells exposed to HL or UV radiations (Supplementary Figure S6). Of note, none of the *psbD* copies were DE in LLLT and the polycistronic copy was even downregulated in HLLT conditions, while all three *psbA* genes encoding the D1:2 isoforms were upregulated in these conditions, suggesting that these stresses induced different damages to the D1 and D2 subunits. Consistently, the expression pattern of the *ctpA* gene, known to encode the D1 protein carboxyl-terminal processing peptidase, mimicked that of D1:2 but not D2 encoding genes, though being much less DE.

A number of other PSII genes, including *psbH-J-L* and *psbE-F1* encoding cyt *b*_559_, were specifically upregulated in the LLUV treatment, like the *psbCD* operon, but more faintly. Interestingly, this parallels the expression of several genes coding for PSII assembly factors. This includes *ALB3*, involved in the integration of precursor D1 proteins (pD1) into the thylakoid membrane (Ossenbühl et al., 2006), *ycf39* that encodes a component of a chl-binding protein complex also including HliC and HliD, which is involved in the delivery of chl *a* to newly synthesized D1 (Knoppová et al., 2014), *ycf48* that encodes a factor participating in both (i) the stabilization of newly synthesized D1 precursors and their subsequent binding to D2–cyt *b*_559_ pre-complexes and (ii) in selective replacement of damaged D1 during PSII repair (Komenda et al., 2012), as well as *psbN* that, by analogy with its homolog in plants, is assumed to be involved in both PSII dimerization during early biogenesis and in PSII repair (Plöchinger et al., 2016; Supplementary Figure S6). Additionally, *deg1*, involved in the degradation of the D1 protein during repair after photoinhibition (Kapri-Pardes et al., 2007) as well as three genes encoding a complex also specifically involved in PSII repair, *ftsH2-3 and psb29* (Bečková et al., 2017), were all specifically upregulated in the LLUV treatment. Altogether, these data suggest that, in our experimental conditions, UV stress is more deleterious for PSII than the other tested treatments in WH7803, and that the full recovery of damaged PSII from UV stress might involve the neo-synthesis of a fairly large number of PSII subunits and not just the reassembly of pre-existing subunits after D1 replacement. In agreement with this hypothesis, several genes involved in key steps of the biosynthesis of chl *a*, which binds at very early steps of PSII assembly and that is required for stabilizing PSII pre-complexes (Komenda et al., 2012), were also more particularly upregulated in response to UV stress. This includes (i) two of the three genes encoding the protoporphyrin IX Mg-chelatase complex (*chlD* and *H*), which catalyzes the insertion of Mg^2+^ into protoporphyrin IX, (ii) *cycI1* that transforms Mg-protoporphyrin IX monomethyl ester into divinyl protochlorophyllide as well as (iii) *cvrA* that transforms divinyl-chlorophyll *a* precursor into monovinyl chl *a* (Islam et al., 2008). Furthermore, the strong UV-induced upregulation of the *pao* gene encoding the pheophorbide *a* oxygenase, responsible for opening of the chlorin macrocycle of pheophorbide *a*, likely indicates that an active chl breakdown occurs in this condition, potentially allowing cells to eliminate molecules of this potentially phototoxic pigment associated to damaged PSII proteins (Hörtensteiner and Kräutler, 2011).

Another stress-specific response related to photosynthesis is the significant induction of genes coding for RuBisCo, most carboxysome subunits as well as all ATPase subunits only in response to HL stress, while being downregulated in response to all other stress conditions (Supplementary Table S2 and Figure 5, section C). This suggests that, although cells seemingly actively respond to the increase in irradiance by reducing the number of photosystems and their light harvesting capacity (as demonstrated by the downregulation of most genes involved in biosynthesis of phycobilisomes, PSI as well as PSII subunits not involved in the maintenance of the D1/D2 turnover), they are able to use the extra photons provided by the increase in light intensity to enhance carbon and energy production. Although less striking, a few photosynthetic genes also seem to be more DE in response to LT stress, including genes coding for the three subunits of NADH dehydrogenase specifically involved in CO_2_ fixation (*cupB*, *ndhD4*/*F4*) as well as *ndhM* and *V*, the two latter subunits being essential for cyclic electron transport around PS I in *Synechocystis* sp. PCC 6803 (Gao et al., 2016; He et al., 2016), the expression pattern of these five genes differing from that of the other Ndh subunits.

### Glucose and Glycogen Metabolism

As previously observed in response to nitrogen deprivation in *Synechocystis* sp. PCC 6803 (Azuma et al., 2011), sugar catabolism pathways were also strongly affected by the different stresses tested in this study. The two glycogen degradation pathways, either through the glycogen phosphorylase (GlgP) or through the isoamylase (GlgX), were indeed both strongly upregulated in LLLT and also slightly (0.5 < log_2_FC ≤ 1) in the LLHL, LLUV and/or HLLT treatments (Figure 6). Conversely, all genes involved in glycogen biosynthesis (*glgA-B-C*) were downregulated in all these conditions, while the two copies of phosphoglucomutase (*pgmA1/A2*), a metabolic branch point between storage and utilization of carbohydrates was also downregulated under UV and LT stresses. Furthermore, most genes of the upper glycolysis (including *pgi*, *fbaA*, and *tpiA*) and the oxidative pentose phosphate (OPP) pathways were slightly downregulated by the different treatments. Conversely, the *eda* gene, recently shown to be essential for the Entner-Doudoroff pathway in *Synechocystis* (Chen et al., 2016) was found to be strongly upregulated in LLHL, LLUV, LLLT, and HLLT treatments (Figure 6 and Supplementary Table S2). While the absence of activation of the OPP pathway is expected since this pathway is mostly active in the dark and in photomixotrophic conditions (Takahashi et al., 2008), these results also tend to support Chen et al. (2016) recent hypothesis that the absence in all marine picocyanobacteria genomes (including WH7803) of orthologs for *Synechocystis* phosphofructokinase genes *pfkA* and *pfkB* likely implies that these organisms are not able to perform the upper part of the glycolysis pathway that transforms glucose-6-phosphate (G6P) into glyceraldehyde-3P (GA3P). The *gap1* gene encoding glyceraldehyde-3-phosphate dehydrogenase type I, the first enzyme of the lower part of the glycolysis pathway, which is shared between the three glucose degradation pathways, was strongly upregulated in both LLHL and LLLT treatments, while its anabolic counterpart *gap2* was unaffected in LLHL and downregulated in LLLT (Figure 6). Moreover, the upregulation of *xfp* that encodes a xylulose 5-phosphate/fructose 6-phosphate (Xu5P/F6P) phosphoketolase that catalyzes the conversion of F6P and inorganic phosphate (Pi) to erythrose 4-phosphate (E4P) and acetyl phosphate (AcP) and/or Xu5P and Pi to glyceraldehyde 3-phosphate (GA3P) and AcP, might provide an additional source of GA3P. Altogether, these results indicate that all stress conditions favored the degradation of glycogen and then glucose into pyruvate mostly via the Entner-Doudoroff pathway, with the concomitant production of ATP, NAD(P)H and biosynthetic precursors for amino acids, nucleotides and fatty acids.

### Lipid Metabolism

Variations in the relative content of glycolipids are well known to be crucial for cell acclimation to temperature changes by modulating membrane fluidity. Thinner membranes (i.e., with a lower fatty acid average length) and/or highly unsaturated membranes, both favoring their fluidity, are indeed commonly observed in cold-adapted organisms (Chintalapati et al., 2007; Iskandar et al., 2013) and in response to cold stress, notably in marine *Synechococcus* (Varkey et al., 2016; Pittera et al., 2018; Breton et al., 2019). Here, although strong correlations and anticorrelations were found between length and saturation levels of the different lipids and blue and turquoise modules, respectively (Figure 4), most genes involved in fatty acid chain biosynthesis, in their insertion into membranes as well as in polar head biosynthesis were not significantly or only slightly DE. This suggests that these processes are likely co-regulated with other temperature-dependent response mechanisms and/or post-transcriptionally regulated. Still, the fatty acyl-ACP reductase, *aar*, was specifically induced in response to LT in HL-acclimated cultures. AAR is involved in alkane biosynthesis, a two-step pathway during which the acyl-acyl carrier protein (ACP) is first reduced to aldehyde by AAR, then the aldehyde is oxidized to alkane by the aldehyde-deformylating oxygenase (ADO; Schirmer et al., 2010). The AAR/ADO pathway was recently shown to be involved in cold stress resistance in *Synechocystis* sp. PCC 6803, since the lack of this pathway provoked an increased cyclic electron flow around PSI in the mutant and a significant growth rate reduction (Berla et al., 2015). Alkanes have thus an essential role in the regulation of the redox balance and reductant partitioning under cold stress.

The most striking observation in this functional category concerns the fatty acid desaturases, which by inserting unsaturations into acyl chains, were shown to play a crucial role in temperature stress response through adjustment of thylakoid fluidity (Ludwig and Bryant, 2012). Consistent with the data of Pittera et al. (2018) showing that after a shift from 22 to 13°C, the level of double unsaturations of the MGDG *sn*-1 chain increased, the delta-12 fatty acid desaturase gene *desA3* was found to be upregulated in response to LT, while it was downregulated in the opposite thermal shift (LLHT). The gene encoding the delta-9-desaturase DesC4, which is absent from warm *Synechococcus* thermotypes (clades II and III; Varkey et al., 2016; Pittera et al., 2018; Breton et al., 2019), also appeared strongly DE at LT but also in other stress conditions. At last, genes coding for DesA2, a delta-12 desaturase mostly found in warm thermotypes (Pittera et al., 2018) and DesC3, a core delta-9 desaturase, were in contrast mostly responsive to UV stress, even though *desA2* was also upregulated in HLLT conditions. Thus, all four WH7803 desaturase genes displayed very different expression patterns in response to the various tested conditions, which might be related to the necessity to finely tune the fluidity of membranes, constituting the matrix for the photosynthetic machinery. Indeed, the membrane unsaturation level plays a role in the assembly and functional regulation of the PSII complex after photoinactivation, notably by influencing the maturation process of the D1 precursor (Kanervo et al., 1997; Mizusawa and Wada, 2012).

### DNA Repair

Many stresses are known to cause DNA damages to bacteria (including cyanobacteria) either directly (e.g., chemicals, gamma or UV-C radiations, etc.) or indirectly through the generation of ROS (e.g., induced by light, UV-A and B radiations, or temperature stress; He and Häder, 2002). Like other marine *Synechococcus*, WH7803 possesses a broad set of DNA repair proteins (Cassier-Chauvat et al., 2016). Some of them are involved in direct DNA damage reversal, such as DNA photolyases or members of the Odt/Ogt family (e.g., YbaZ, a 6-*O*-alkylguanine DNA alkyltransferase likely involved in G:C to A:T transversions; Mazon et al., 2009). WH7803 possesses four distinct putative photolyases, one of them being synthesized by an operon of two small genes, the first one coding for the FAD-binding domain (CK_00001541) and the second one for the photolyase domain (CK_00001540). Three of these photolyases were strongly upregulated in response to most stresses and more specifically to LLUV, while the fourth one (CK_00001460), whose C-terminal domain is only distantly related to the typical DNA photolyase domain, showed only a faint response in LLUV and HLLT conditions. In contrast, the *ybaZ* gene was most strongly upregulated in response to LLHL and LLLT. Altogether, it seems that the WH7803 genes involved in direct DNA damage reversal are activated in response to distinct stresses.

WH7803 also possesses many genes potentially involved in SOS response (Supplementary Table S2 and Supplementary Figure S7; see also Blot et al., 2011; Cassier-Chauvat et al., 2016). In *E. coli*, this pathway constitutes an essential response to stress-induced DNA damages, starting by the formation by either RecBCD or RecFOR complexes of single-strand DNA (ssDNA), recognized by RecA, which then catalyzes the auto-proteolysis of the SOS regulon repressor LexA (see Baharoglu and Mazel, 2014 for a review). In contrast, in *Synechocystis* sp. PCC 6803, the latter protein has been shown to regulate carbon assimilation and cell motility but surprisingly not DNA repair (Domain et al., 2004; Kizawa et al., 2016). Accordingly, this strain lacks several DNA repair genes present not only in *E. coli* but also in marine *Synechococcus* spp. (namely *mutY* and *recB*, *C*, *J*, and *Q*). It is therefore quite possible that marine *Synechococcus* display an SOS response, regulated by LexA, a hypothesis supported by the fact that in WH7803, both *recA* and *lexA* were strongly upregulated in response to UV shifts (both LLUV and HLUV) and more faintly to low temperature (either LLLT or HLLT) and high light (LLHL) shifts. Interestingly, the biosynthesis of the RecBCD and RecF(O)R complexes, recognizing double-strand breaks and gaps in one DNA strand, respectively, did not seem to occur in the same conditions in WH7803. While the former encoding genes were slightly upregulated at LT, the latter were mostly upregulated during the LLUV shift, suggesting that these stresses induce different DNA damages. Of note, *recO* displayed a different expression pattern than *recF* and *recR*, questioning whether RecO is truly part of the same complex as RecF-R in marine *Synechococcus*, especially since WH7803 RecO-like sequence is less similar to *E. coli* RecO than the corresponding RecF and RecR counterparts. It is also interesting to note that *dprA*, which displaces SSB, loads RecA onto ssDNA, protects ssDNA from nucleases (Hovland et al., 2017) and was previously suggested to belong to the LexA regulon in WH7803 (Blot et al., 2011), is specifically and strongly induced under UV stress.

Additionally, the nucleotide excision repair (NER) pathway, driven by UvrABC, which allows the repair of lesions in double-stranded DNA (Sancar and Rupp, 1983), was also activated in WH7803 (Supplementary Figure S7). Genes coding for the UvrA-B complex, which recognizes the DNA lesion, were most strongly upregulated in response to LLUV while UvrC, which makes incisions on both sides of the lesion before the UvrD helicase removes the ssDNA carrying the lesion (Kumura et al., 1985), also responded to the HLLT shift. Surprisingly, the gene encoding UvrD, which removes the ssDNA carrying the lesion, showed no differential expression whatever the stress, suggesting a post-transcriptional regulation. The combined action of the DNA polymerase I and a ligase then fills the gap (Husain et al., 1985). In WH7803, both genes were upregulated by both UV and LT stresses.

The last pathway, involving translesion synthesis (TLS) polymerases, is known to be activated only when the SOS-inducing signal persists and allows highly mutagenic bypass of DNA lesions that are not effectively passed through by the standard replicative DNA polymerase Pol III (Patel et al., 2010). In WH7803, the only polymerase potentially involved in this pathway is polymerase V, encoded by the *umuCD* operon, which like *recA* was most strongly upregulated under UV conditions and also responded to the HLLT shift. This suggests that UV and HLLT constitute the most damaging stresses for DNA that can still be repaired but at the expense of an increased frequency of spontaneous mutations.

### Compatible Solutes and Osmoregulation

Besides their role as osmolytes in salt-stressed cells, compatible solutes are also known to directly protect enzymes and membranes against denaturation caused by other environmental stresses and notably high or low temperatures (Diamant et al., 2003; Hincha and Hagemann, 2004; Pade and Hagemann, 2015). *Synechococcus* sp. WH7803 possesses all genes (*ggpP*, *S* and *ggtA-D*) involved in the synthesis and uptake of the heteroside glucosylglycerol (GG), and its ability to accumulate GG, considered as the main compatible solute in marine picocyanobacteria, has been experimentally verified in this strain (Scanlan et al., 2009). Still, all of these genes were downregulated in all stress conditions and showed minimal variations during the L/D cycle, except *ggpP*, displaying upregulation during the day with a peak at noon. Although WH7803 was found to accumulate a considerable amount of sucrose, also known to be involved in osmoregulation, the gene coding for the sucrose phosphate synthase and phosphatase fusion protein (*sps-sds*) was also downregulated in stress conditions and the same is true for most genes involved in synthesis (*gbmt1-2*) and transport/re-uptake (*proW-X*) of glycine betaine (GB), which was shown to be accumulated by WH7803 cells (Scanlan et al., 2009). In contrast, the gene coding for an additional mono-subunit GB uptake system (*betP*) was specifically upregulated under LT (LLLT and HLLT), while both genes (*gpgP*, *S*) potentially involved in glucosyl glycerate (GGA) synthesis, a negatively charged compatible solute, were specifically upregulated in response to HL stress, potentially explaining why it was not detected in extracts of WH7803 grown in standard conditions (Scanlan et al., 2009). Interestingly, genes involved in osmolyte synthesis and transport were also quite differently regulated by the L/D cycle with most genes involved in GG synthesis and transport showing only faint diel variations, GGA genes being most strongly expressed during the day, while GB genes displayed the most robust diel oscillation with a peak at the L/D transition and still a strong expression in the first part of the dark period.

In summary, although WH7803 is able to accumulate GG, possibly GGA and to take up GG, sucrose and GB, these compounds appear to be involved in osmoregulation at different times of the cell cycle and to respond to distinct environmental conditions.

### Oxidative Stress Response and Photoprotection

#### General Oxidative Stress Response

One of the particularities of photosynthetic organisms is that in response to various environmental stresses, the photosynthetic electron transport can sometimes outpace the rate of electron consumption by CO_2_ fixation, leading to a rapid increase of intracellular ROS, which can produce deleterious effects on the cellular machinery, including DNA, lipids, proteins and notably PSII reaction centers (Nishiyama et al., 2006; Takahashi and Murata, 2008; Latifi et al., 2009). In WH7803, a different set of genes involved in ROS protection and detoxification mechanisms was DE in response to distinct stresses, suggesting that like in freshwater cyanobacteria, the different systems may function under a particular condition (Perelman et al., 2003). Among the two WH7803 genes coding for superoxide dismutase (SOD), which catalyzes the dismutation of the superoxide (O_2_^–^) radical into O_2_ and hydrogen peroxide (H_2_O_2_), only *sodB* encoding the Fe-SOD was significantly upregulated in LLHL and LLUV, while *sodC*, encoding the Cu/Zn-SOD was only faintly upregulated in HLLT. The gene coding for the glutathione peroxidase (Gpx), catalyzing the decomposition of H_2_O_2_ to water and O_2_, was downregulated in response to LT stress, while for comparison the two glutathione peroxidases present in *Synechocystis* sp. PCC 6803 were shown to be upregulated in response to HL and high salinity and to be essential for the protection of membranes against lipid peroxidation (Gaber et al., 2004; Cameron and Pakrasi, 2010). Similarly, the catalase-peroxidase, KatG, only present in a few picocyanobacterial strains and also acting on H_2_O_2_, was also downregulated in WH7803 but in all stressful conditions. In contrast, three out of the four peroxiredoxins (*ahpC*, *prxQ1*, and *prxQ2*), also potentially involved in H_2_O_2_ detoxification, were upregulated, the latter two responding to most stressful conditions, while *ahpC*, encoding a 2-Cys peroxiredoxin, seemed to be more specific to HL. The differential expression of these genes may be in part because catalases mainly detoxify high levels of H_2_O_2_, while peroxiredoxins were shown to rather scavenge low levels of this compound (Stork et al., 2005).

Thioredoxins (TRX) and glutathiones (GSH) are also known to play a critical role in the maintenance of the redox homeostasis and protection from ROS in cyanobacteria (Cameron and Pakrasi, 2010; Sánchez-Riego et al., 2016). Here, among the genes involved in the synthesis of these thiol molecules, the two gene copies encoding glutathione *S*-transferases (*gst*), were only transiently upregulated in response to LLUV stress, while two out of the six *gst* genes present in *Synechocystis* sp. PCC 6803, *sll1545* and *slr0236*, both homologs of CK_00000203, have been shown to operate in the protection against stresses such as high light and H_2_O_2_ (Kammerscheit et al., 2019). Additionally, the *Synechococcus* core *trxB* gene, involved in the NADPH-dependent thioredoxin reductase C (NTRC) system (Scanlan et al., 2009), was the only thiol-encoding gene to be significantly upregulated in all stressful conditions. Interestingly, NTRC as well as sulfiredoxin, which in WH7803 displayed a similar gene expression pattern, have been shown in plants and *Anabaena* sp. PCC 7120 to be involved in the protection of the photosynthetic apparatus against oxidative stress damages through an effective reduction of 2-Cys Prx (Sánchez-Riego et al., 2016). Altogether, it seems that at least in the tested conditions, the ROS protection and detoxification systems in WH7803 resemble more to the ones described in PCC 7120 and in plants, formed by NTRC, 2-Cys Prx and sulfiredoxin, than to the ones of PCC 6803 that lacks both NTRC coding gene and sulfiredoxin and which strategy mainly relies on a high peroxidase/catalase activity (Sánchez-Riego et al., 2016). However, we cannot exclude that in other (e.g., more stressful) conditions, the peroxidase/catalase system could be activated for ROS detoxification in WH7803.

Another more unusual antioxidant system detected in this study is the cystein desulfurase SUF system. It is responsible for Fe–S cluster biosynthesis in conditions of Fe limitation or oxidative stress in many bacteria (Nodop et al., 2008; Bolstad and Wood, 2010; Dai and Outten, 2012) and constitutes the major pathway for Fe–S cluster assembly in cyanobacteria (Ayala-Castro et al., 2008; Banerjee et al., 2017). In marine picocyanobacteria, the *sufBCD* operon, which is highly conserved in cyanobacteria, is adjacent to the *sufR* transcriptional repressor and all sequenced picocyanobacterial genomes also possess a group I *sufS* (CK_00000030), homologous to the *Synechocystis* sp. PCC 6803 group I NifS-like cysteine desulfurase, Slr0387 (Tirupati et al., 2004). Here, the gene coding for the SufE protein, involved in the sulfur release from cysteine and that can stimulate the cysteine desulfurase activity of SufS, was found to be quite strongly upregulated in LT, while *sufBCDS* were mostly upregulated in LLLT but also in LLHL and late LLUV stresses. Thus, by analogy with *Anabaena* sp. PCC 7120, in which the SufS protein was suggested to enhance oxidative stress tolerance by decreasing the intracellular ROS (Banerjee et al., 2017), the SUF system could also be an important antioxidant system in marine picocyanobacteria in particular under LT stress. In this context, it is also worth noting that genes coding for two manganese transporters, the high-affinity MntABC transport system and another ABC-type Mn^2+^/Zn^2+^ transport system (SynWH7803_0988-90) were particularly upregulated under LT stresses, suggesting that like in *Anabaena* sp. PCC 7120, Mn could also play a role in protection against ROS due to its antioxidative properties or involvement as a cofactor in several antioxidative enzymes (Kaushik et al., 2015).

#### Photoprotection

In cyanobacteria several mechanisms are involved in the dissipation of excess light as heat in order to limit the production of ROS. While WH7803 lacks the iron stress-induced protein IsiA, potentially involved in light energy dissipation (Havaux et al., 2005), as well as a plastoquinol terminal oxidase (PTOX), an oxidase which could be involved in the removal of electrons from the intersystem photosynthetic electron transport chain (Bailey et al., 2008), this strain possesses several other mechanisms potentially involved in photoprotection and conserved in most cyanobacteria, as detailed below.

Although the exact role of high-light-inducible proteins (HLIPs) is not fully understood, the four *hli* genes of *Synechocystis* sp. PCC 6803 seem to respond differentially to various stresses and their products are thought to prevent photodamages to chl protein complexes by quenching deleterious singlet excited states of chl *a* and/or singlet oxygen (Hirayama et al., 1994; Huang et al., 2002). More specifically, two of them, HliC and D, were recently shown to bind chl and β-car in *Synechocystis* sp. PCC 6803 that could be used for chl recycling (Niedzwiedzki et al., 2016; Shukla et al., 2018). Among the nine *hli* genes present in WH7803, two were highly expressed under most stress conditions, four (including CK_00000050, the closest homolog of PCC 6803 HliC) were more specifically induced by UV and HL treatments, two others were only faintly DE in response to stress, while the last one (CK_00001058, corresponding to the closest homolog of PCC 6803 HliD) did not respond to any monitored stress (Supplementary Table S2). Interestingly, while most *hli* genes were upregulated during the day and downregulated at night, the latter copy, a core and quite well-conserved picocyanobacterial gene, was specifically induced during the night. This somehow recalls the L/D expression patterns of *Prochlorococcus* MED4 *hli4* and *hli11*, which were also found to peak during the night (Zinser et al., 2009).

Photoprotective responses in cyanobacteria also involve the thermal dissipation of excess light energy captured by the light harvesting antenna by the orange carotenoprotein (OCP; Wu and Krogmann, 1997; Kirilovsky and Kerfeld, 2016). The *ocp* gene is part of an operon of three genes, also including the β-carotene ketolase (*crtW*), involved in the synthesis of the keto-carotenoid bound to the OCP protein and the fluorescence recovery protein (FRP), responsible for acceleration of the detachment of the OCP from the PBS complex and subsequent deactivation of the OCP (Thurotte et al., 2017). In *Synechocystis* sp. PCC 6803, the OCP was found to be strongly induced by various stresses that exacerbate photodamage and/or prolongate the excited state of antenna chromophores, notably HL (Hihara et al., 2001), LT (Kerfeld et al., 2017) and oxidative stresses (Shrivastava et al., 2016), but seems to be constitutively expressed and to remain at low concentration in non-stressed conditions (Gwizdala et al., 2011). Here, these genes were indeed only moderately induced during the first part of the light period but were among the most strongly induced genes in response to light stress (HL and UV) in LL-acclimated cultures, while they were in contrast more upregulated by LT in HL- than in LL-acclimated cultures. Altogether, the expression pattern of this operon is thus consistent with a potential synergic effect between HL and LT, as discussed above.

Besides the abovementioned beta-carotene ketolase, several genes involved in carotenoid biosynthesis pathways were also upregulated in response to several stresses. This included *crtPQ* (LLHL and LLUV), involved in the biosynthesis of lycopene, and *crtL*-b (LLLT and HLLT) encoding a cyclase enzyme, which turns lycopene into β-carotene (Supplementary Figure S8). The latter pigment, which is mainly located in the molecular neighborhood of the chl *a* molecules bound to reaction centers and acts as an antioxidant (Telfer, 2005), has previously been shown to be involved in the cold stress response (Pittera et al., 2014). Since the β-carotene to chl *a* ratio decreased in these conditions (Supplementary Figure S2D), the upregulation of *crtL*-b suggests that the rate of β-carotene breakdown by ROS exceeded its synthesis rate during the stress period. Interestingly, the *crtL-b* gene was recently shown to display the highest number of substitutions specific of the cold-adapted clades I and IV (Doré et al., under revision), and could thus play a crucial role in the adaptation to cold temperature in marine *Synechococcus*. The likely increase of β-carotene synthesis can also be linked to its role of precursor in the *Synechococcus* xanthophyll pathway. Indeed, *crtR*, coding for an hydroxylase involved in zeaxanthin biosynthesis, was upregulated in response to HL and UV stress, consistent with the increase of the ß-cryptoxanthin and zeaxanthin to chl *a* ratios in these conditions (Supplementary Figures S2B,C). Although the role of zeaxanthin has never been formerly demonstrated, it is likely that it affords photoprotection during stress periods.

At last, a gene coding for a carotenoid cleavage dioxygenase (*diox1*), catalyzing the oxidative cleavage of apo-carotenoids was strongly upregulated in all stressful conditions (Cui et al., 2012). As previously suggested for freshwater cyanobacteria (Hihara et al., 2003; Scherzinger et al., 2006), these apo-carotenoids could arise from photodestruction processes mediated by ROS, and cleavage products (retinal or retinal-like compounds) could be involved in stress signaling in WH7803.

## Conclusion

This work constitutes the first comprehensive study of the response of a ubiquitous marine picocyanobacterium to various stresses, taking into account the light history of the cells. It allowed us to identify the common and specific responses of this organism to a variety of ecologically relevant environmental conditions. Most genes were found to respond to a subset of the tested conditions (Supplementary Figure S5), with the most stressful treatments (LLHL, LLUV, LLLT, and HLLT) notably triggering a strong induction of numerous chaperone- and protease-encoding genes and of the psbA gene copies encoding the D1:2 isoform of the D1 protein, while most genes involved in the neosynthesis of PSII were in contrast downregulated. Yet, a number of genes were specifically up- or down-regulated in only one treatment or one stress type. Although LLLT and HLLT shifts globally triggered the strongest transcriptomic response (Figures 3A,B), fairly few genes with known function were specifically induced by these treatments. This includes genes coding for NADH dehydrogenase subunits involved in CO2 fixation (*cupB*) and cyclic electron transport around PSI (*ndhV*), some genes of the glycogen degradation pathway, as well as *betP* encoding a component of the glycine betaine uptake system. Concerning the specific response to the HL treatment, the most striking one was the induction of genes encoding RuBisCo as well as most carboxysome and ATPase subunits, while these genes were downregulated in other conditions, suggesting that the HL shift boosted growth of the culture rather than slowed down cell metabolism. The UV treatment induced a few specific protease-encoding genes (*clpP1-4* and *clpC*), the *dprA* gene involved in repair of single-stranded DNA, one *psbD* gene copy and several other genes involved in the neo-synthesis of PSII subunits, as well as several *hli* genes, possibly coding for proteins involved in providing chl *a* molecules to newly synthesized PSII subunits (Niedzwiedzki et al., 2016). The latter treatment appears to be the most damaging condition for PSII reaction centers when WH7803 cells were pre-acclimated to LL, while cells acclimated to HL seemed to be better prepared to withstand this stress, likely due to a faster turnover of the D1 protein. In contrast, a pre-acclimation to HL seemed to enhance the effects of LT on the WH7803 transcriptome compared to cells pre-acclimated to LL. By analogy with a previous report showing a synergic effect of a prior exposure to HL on the response of WH7803 cells to oxidative stress (Blot et al., 2011), we hypothesize that the deleterious effects of ROS could also be amplified by the slowing down of the cell metabolism at LT, causing a slower turnover of the D1 protein of PSII. More generally, our results suggest that the sensitivity of the D1 protein to oxidative stress plays a central role in the response of these organisms to variations in environmental conditions affecting cell metabolism. The observed synergic effect between LT and HL might have important implications for the dynamics of natural *Synechococcus* populations in the field, where cells are often subjected to concomitant variations of temperature and irradiance, notably in upwelling areas or during seasonal mixing events. In these circumstances, this effect could negatively impact the competitiveness of *Synechococcus* cells with regard to other phytoplanktonic groups, such as picoeukaryotes or diatoms that often predominate in cold mixed waters (Uysal and Köksalan, 2006; Schmidt et al., 2020).

This study also highlights several specificities of the *Synechococcus* stress response compared to well-studied freshwater model cyanobacteria, such as *Synechocystis* sp. PCC 6803, therefore justifying the interest of studying marine models. Altogether, the availability of the unique set of 154 transcriptomes described here, notably including L/D cycles at two temperatures, should be particularly useful to better interpret the ever-increasing metatranscriptomic data generated from a variety of ecological niches in the marine ecosystem and at different times of the day.

## Data Availability Statement

Transcriptomic data supporting the conclusions of this article are available as raw and processed data in the SRA (SRP251300) and GEO (GSE146246) databases, respectively, and sample descriptions and accession numbers per biological replicate are available in Supplementary Table S3. Differential expression levels and associated statistics as well as genomic features of the *Synechococcus* sp. WH7803 strain, such as core genes and predicted cyanorons, are available in Supplementary Table S2.

## Author Contributions

CSi, MR, and LG designed the experiments. NN, HD, JP, MR, CSi, FP, and LG collected the samples and performed the physiological measurements. NN and MR extracted the RNA. NN, MR, CSt, and LG developed the protocol for RNA library preparation. UG, HD, LG, GL, and EC developed and ran the RNAseq analysis pipeline. LB-G, MH, and UG integrated the genome and transcriptome data into the JBrowse genome browser available through the Cyanorak v2.1 information system. MC, AS, FP, LG, and UG manually annotated genes and pathways. JH, UG, HD, LG, and DE performed the comparative transcriptomic analyses. UG, JH, FP, and LG made the figures. UG, HD, FP, and LG interpreted the results. All the authors contributed to the preparation of the manuscript, read and approved the final manuscript.

## Conflict of Interest

The authors declare that the research was conducted in the absence of any commercial or financial relationships that could be construed as a potential conflict of interest.

## Footnotes

1 http://roscoff-culture-collection.org/

2 www.sb-roscoff.fr/cyanorak

3 https://www.genome.jp/kegg/

## Supplementary Material

FIGURE S1 Description of the experimental design used in the present study. Insert in panel B illustrate the light curve used for the L/D cycle and sampling points are indicated by black arrows. Ct, controls; HL, high light; UV, ultraviolet; LT, low temperature; HT, high temperature; R, recovery; L/D, light dark; μE, μEinstein m^–2^ s^–1^.

FIGURE S2 Variations of the major liposoluble pigment ratios in *Synechococcus* sp. WH7803 acclimated to low (LL, 20 μE m^–2^ s^–1^ or high light (HL, 250 μE m^–2^ s^–1^). **(A,D)** β-carotene to chlorophyll (chl) *a*, **(B,E)** β-cryptoxanthin to chl *a* and **(C,F)** Zeaxanthin (zea) to chl *a* ratios in response to shifts to **(A–C)** HL, ultraviolet radiations (UV-A 3 W m^–2^, UV-B 0.3 W m^–2^), **(D–F)** low temperature (LT: shift from 22 to 13°C) or high temperature (HT; shift from 22°C to 30°C). The top gray banners indicate the light acclimation condition. These data are based on at least three independent experiments.

FIGURE S3 Variations of phycobiliprotein fluorescence emission ratio in *Synechococcus* sp. WH7803 acclimated to low (LL, 20 μE m^–2^ s^–1^ or high light (HL, 250 μE m^–2^ s^–1^). **(A,C)** Phycoerythrin (PE) to phycocyanin (PC) ratio after a shift **(A)** to high light or ultraviolet radiations (UV-A 3 W m^–2^, UV-B 0.3 W m^–2^), and **(C)** to low temperature (LT: shift from 22 to 13°C) or high temperature (HT; shift from 22 to 30°C). **(B,D)** Same for the PC to terminal acceptor (TA) ratio. The dashed line indicates the time (6h) at which cultures submitted to light stresses were shifted back to their initial light conditions to study recovery. The top gray banners indicate the culture acclimation condition. These data are based on at least three independent experiments. Ct, control.

FIGURE S4 Number of differentially expressed (DE) genes in *Synechococcus* sp. WH7803 at 27°C as compared to 21°C. The top white and black banners indicate light and dark periods. Data are based on four biological replicates. Abbreviation in *x*-axis correspond to ‘sampling time’. Red and blue colors indicate up- and down-regulated genes, respectively.

FIGURE S5 Distribution of genes in the different shift conditions for the three modules as determined using the UpSet R package. Modules **(A)** Yellow, **(B)** Blue, **(C)** Magenta. Only genes with a |Log2FC| ≥ 1 and padj ≤ 0.05 in at least one condition have been taken into account.

FIGURE S6 Photosystem II neosynthesis and repair. **(A)** Neosynthesis occurs in a stepwise manner and requires several assembly factors (ALB3, Psb28, Psb27, Sll0933-like protein) as well as the D1 protein carboxyl-terminal processing peptidase CtpA (ovals), which transiently interact to mediate the various PSII assembly steps. Note that *Synechococcus* sp. WH7803 possesses an homolog of *Synechocystis* sp. PCC 6803 Sll0933 (CK_00000788) but no homolog of PratA, so the latter factor is probably absent, as indicated by the dotted oval. **(B)** Repair after UV photodamage. When the active photosystem is photodamaged the Mn_4_CaO_5_ cluster (not shown) and the extrinsic proteins (PsbO, PsbU, PsbV, CyanoP and CyanoQ) as well as the CP43 complex leave the complex. The damaged D1 (and D2?) protein(s) is (are) then removed by the FtsH2/3 hetero- oligomeric complex. The Ycf48 protein binds to PS II, whilst the CtpA protease processes the carboxyl terminal extension of pD1. The Psb27 protein associates with CP43, which attaches to PSII after the replacement D1 (and D2?) proteins. Psb27 leaves the complex and the Mn_4_CaO_5_ cluster and extrinsic proteins in turn bind to the PSII monomer, before its dimerization (not shown). PSII subunits or assembly factors, which genes were found to be significantly upregulated (log2FC ≥ 1) or downregulated (log2FC ≤ −1) in response to the LLUV treatment (see Supplementary Table S2), are indicated in red and green, respectively, while others are shown in black. PSII subunits are only designated by letters or numbers at the ends of their respective Psb protein names (e.g., “I” and “30” for PsbI and Psb30, respectively), except for the core PSII subunits, which are designated by their common names (D1, D2, CP43, and CP47). pD1 and pD2 are the precursors forms of D1 and D2, respectively. Note that D2 and pD2 are indicated with a question mark because it is usually considered that only D1 is actively replaced during the PSII repair process. Adapted from Nickelsen and Rengstl (2013) and Mabbitt et al. (2014).

FIGURE S7 Representations of the main DNA repair pathways induced during the SOS response. Yellow stars represent DNA lesions. The full description of each protein is available in Supplementary Table S2.

FIGURE S8 Expression of *Synechococcus* sp. WH7803 genes involved in carotenoid biosynthesis in either LLHL and LLUV **(left)** or LLLT and/or HLLT **(right)**. Upregulated genes are indicated in red, downregulated ones are in green, while not significantly differentially expressed genes are in black. Product abbreviations: GGPP, Geranylgeranyl pyrophosphate; FPP, Farnesyl pyrophosphate. Enzyme names: *crtE, GGPP synthase, crtB*, phytoene synthase*; crtP*, phytoene dehydrogenase; *crtQ*, 9 9′-di-*cis*-zeta-carotene desaturase; *crtL-b*, lycopene beta cyclase; *crtR*, beta-carotene hydroxylase.

TABLE S1 Description of biological, physical or chemical traits used in this study for correlation analyses with the eigenvalue of each WGCNA module. All data were generated in this study except lipid data, which were previously reported in Pittera et al. (2018). Note that only parameters exhibiting a significant correlation with at least one of the WGCNA modules or submodules are shown in Figure 4.

TABLE S2 Inventory and description of *Synechococcus* sp. WH7803 genes and their expression patterns in the different tested conditions: HL (yellow background), UV (purple background), LT (blue background), HT (red background), L/D cycle 21°C vs. 6 h (light green), L/D cycle 27°C vs. 6 h (medium green), L/D cycle 27°C vs. 21°C (dark green). Gene expression values are shown as heatmap with in red the upregulated genes and in green, the downregulated genes. The first column corresponds to the clustering based on expression values within each WGCNA module. CK, Cyanorak; WGCNA, Weighted correlation network analysis; PC, phycocyanin; PE, phycoerythrin; TA, terminal acceptor; F_V_/F_M_, PSII maximal photochemical yield; β-car, β-carotene; chl, chlorophyll; β-crypto, β-cryptoxanthin; zea, zeaxanthin; MGDG, monogalactosyldiacylglycerol; DGDG, digalactosyldiacylglycerol; SQDG, sulfoquinovosyldiacylglycerol; PG, phosphatidylglycerol; VIP, value importance in projection; kTotal, connectivity of each gene within the WGCNA network; kWithin, connectivity of each gene within its WGCNA module; kOut, connectivity of each gene outside its WGCNA module; kDiff, difference between kWithin and kOut.

TABLE S3 Description and GEO accession numbers of the transcriptomic samples analyzed in this study.
