# Supplementary material for: Synergic Effects of Temperature and Irradiance on the Physiology of the Marine Synechococcus Strain WH7803
Source: Front Microbiol. 2020 Jul 24;11:1707. doi: 10.3389/fmicb.2020.01707 (PMC7393227; doi:10.3389/fmicb.2020.01707)
Supplement: FIGURE S1 — Description of the experimental design used in the present study. Insert in panel B illustrate the light curve used for the L/D cycle and sampling points are indicated by black arrows. Ct, controls; HL, high light; UV, ultraviolet; LT, low temperature; HT, high temperature; R, recovery; L/D, light dark; μE, μEinstein m–2 s–1. [file Presentation_1.PPTX]

## Slide 1
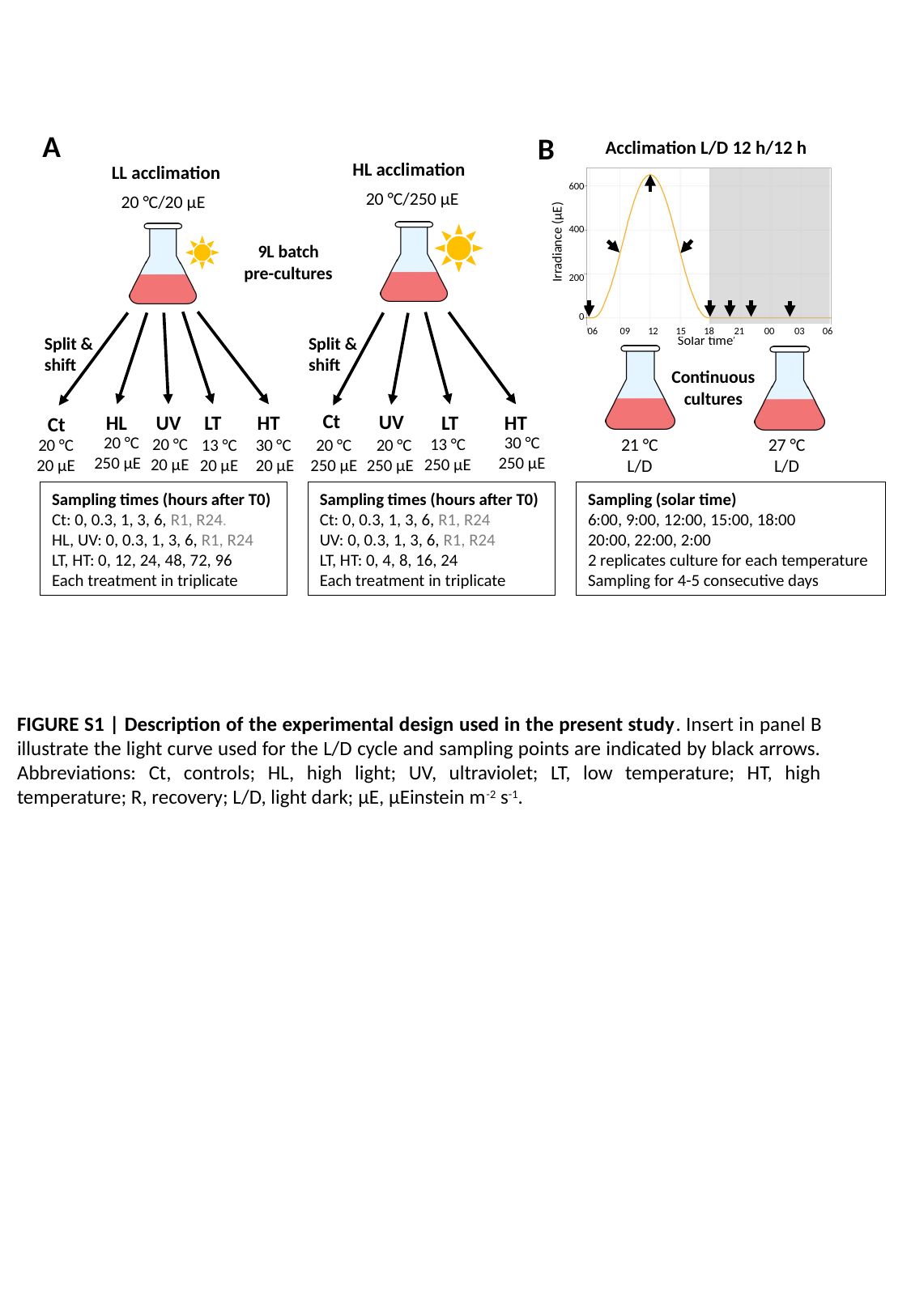

A
B
Acclimation L/D 12 h/12 h
HL acclimation
LL acclimation
600
400
200
0
20 °C/250 µE
20 °C/20 µE
Irradiance (µE)
9L batch pre-cultures
06 09 12 15 18 21 00 03 06
Split &
shift
Split &
shift
Solar time
Continuous cultures
Ct
UV
LT
HT
HL
UV
LT
HT
Ct
20 °C
250 µE
30 °C
250 µE
20 °C
20 µE
13 °C
250 µE
21 °C
L/D
27 °C
L/D
20 °C
250 µE
13 °C
20 µE
20 °C
20 µE
20 °C
250 µE
30 °C
20 µE
Sampling times (hours after T0)
Ct: 0, 0.3, 1, 3, 6, R1, R24.
HL, UV: 0, 0.3, 1, 3, 6, R1, R24
LT, HT: 0, 12, 24, 48, 72, 96
Each treatment in triplicate
Sampling times (hours after T0)
Ct: 0, 0.3, 1, 3, 6, R1, R24
UV: 0, 0.3, 1, 3, 6, R1, R24
LT, HT: 0, 4, 8, 16, 24
Each treatment in triplicate
Sampling (solar time)
6:00, 9:00, 12:00, 15:00, 18:00
20:00, 22:00, 2:00
2 replicates culture for each temperature
Sampling for 4-5 consecutive days
FIGURE S1 | Description of the experimental design used in the present study. Insert in panel B illustrate the light curve used for the L/D cycle and sampling points are indicated by black arrows. Abbreviations: Ct, controls; HL, high light; UV, ultraviolet; LT, low temperature; HT, high temperature; R, recovery; L/D, light dark; µE, µEinstein m-2 s-1.

## Slide 2
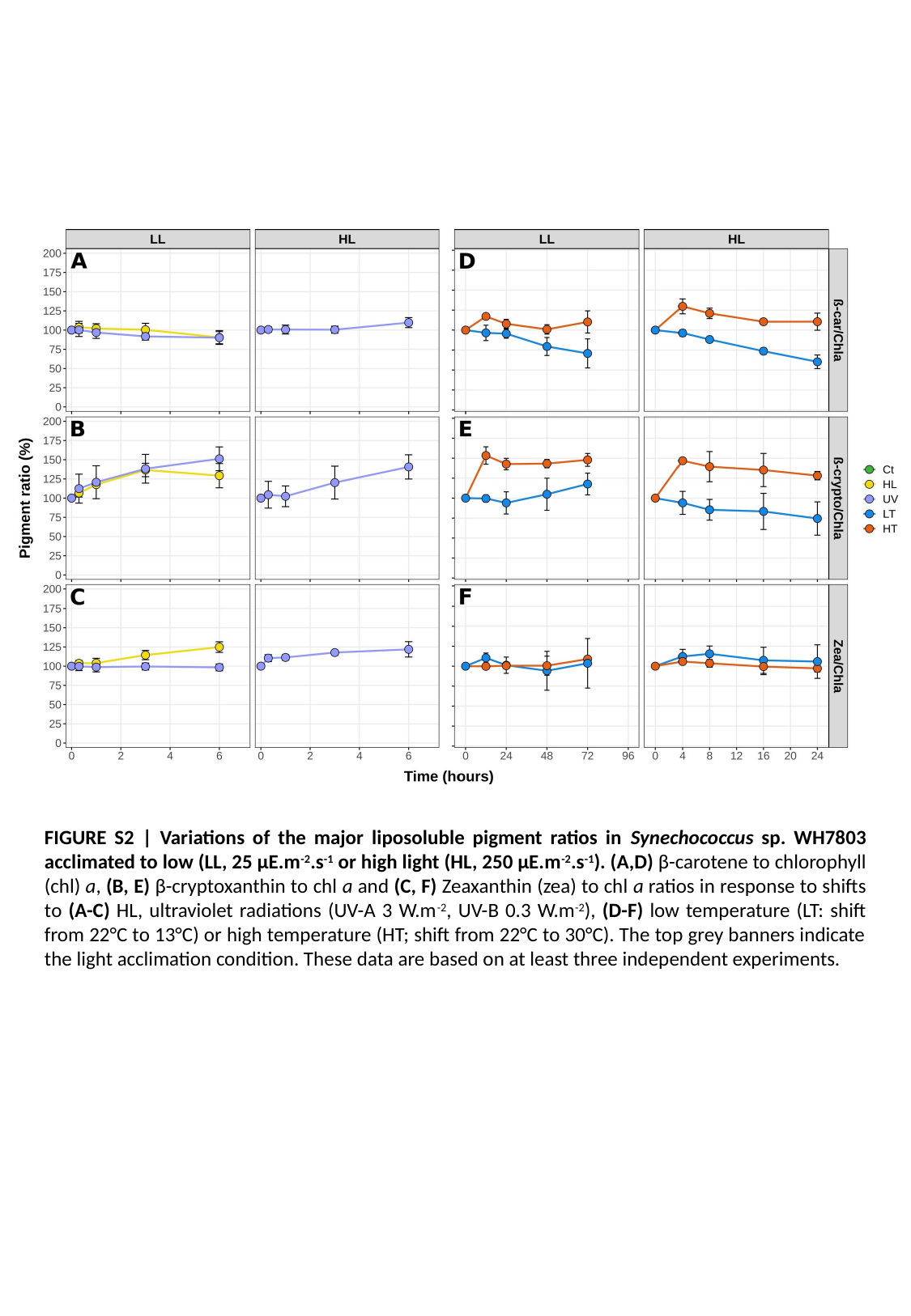

FIGURE S2 | Variations of the major liposoluble pigment ratios in Synechococcus sp. WH7803 acclimated to low (LL, 25 µE.m-2.s-1 or high light (HL, 250 µE.m-2.s-1). (A,D) β-carotene to chlorophyll (chl) a, (B, E) β-cryptoxanthin to chl a and (C, F) Zeaxanthin (zea) to chl a ratios in response to shifts to (A-C) HL, ultraviolet radiations (UV-A 3 W.m-2, UV-B 0.3 W.m-2), (D-F) low temperature (LT: shift from 22°C to 13°C) or high temperature (HT; shift from 22°C to 30°C). The top grey banners indicate the light acclimation condition. These data are based on at least three independent experiments.

## Slide 3
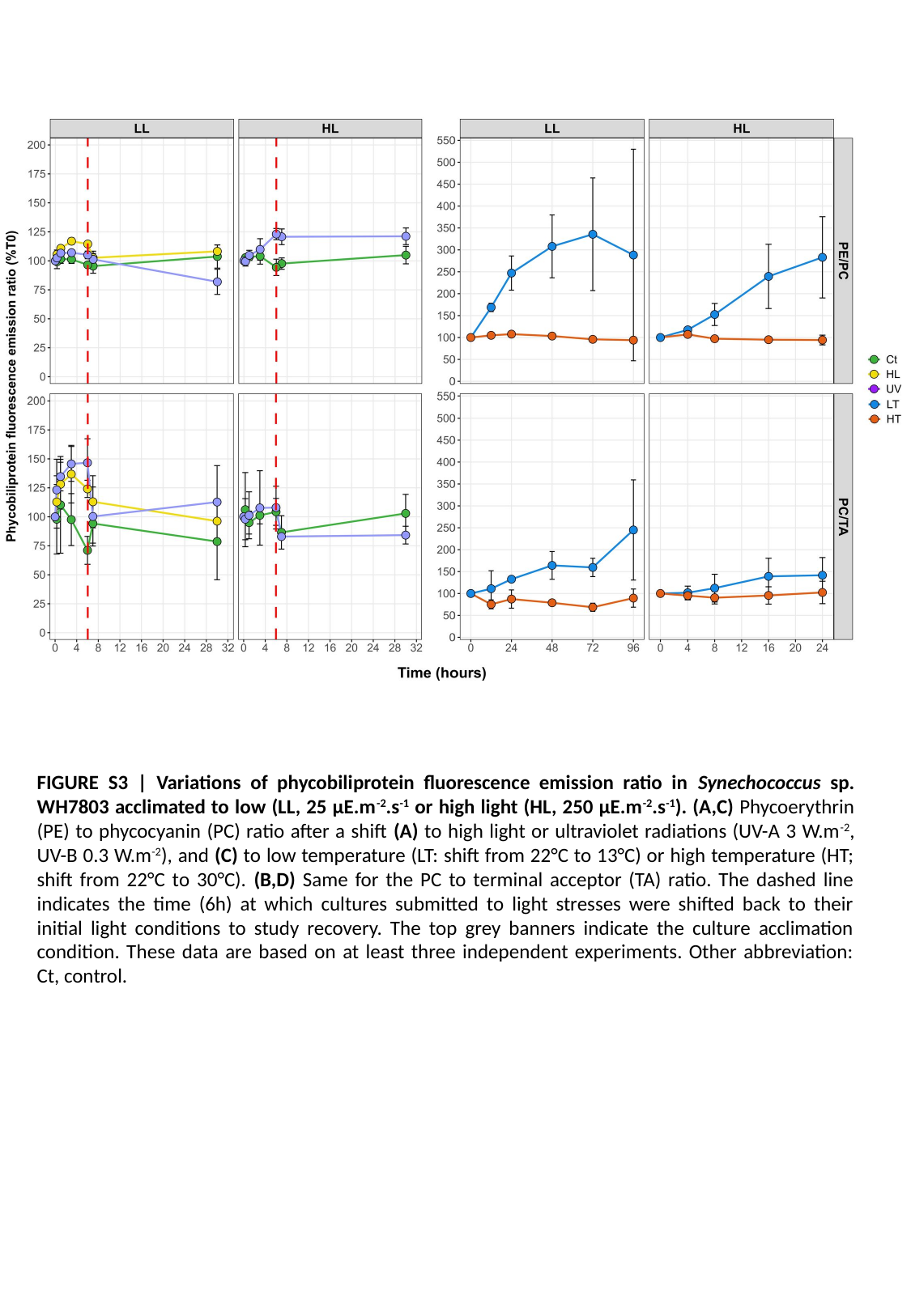

FIGURE S3 | Variations of phycobiliprotein fluorescence emission ratio in Synechococcus sp. WH7803 acclimated to low (LL, 25 µE.m-2.s-1 or high light (HL, 250 µE.m-2.s-1). (A,C) Phycoerythrin (PE) to phycocyanin (PC) ratio after a shift (A) to high light or ultraviolet radiations (UV-A 3 W.m-2, UV-B 0.3 W.m-2), and (C) to low temperature (LT: shift from 22°C to 13°C) or high temperature (HT; shift from 22°C to 30°C). (B,D) Same for the PC to terminal acceptor (TA) ratio. The dashed line indicates the time (6h) at which cultures submitted to light stresses were shifted back to their initial light conditions to study recovery. The top grey banners indicate the culture acclimation condition. These data are based on at least three independent experiments. Other abbreviation: Ct, control.

## Slide 4
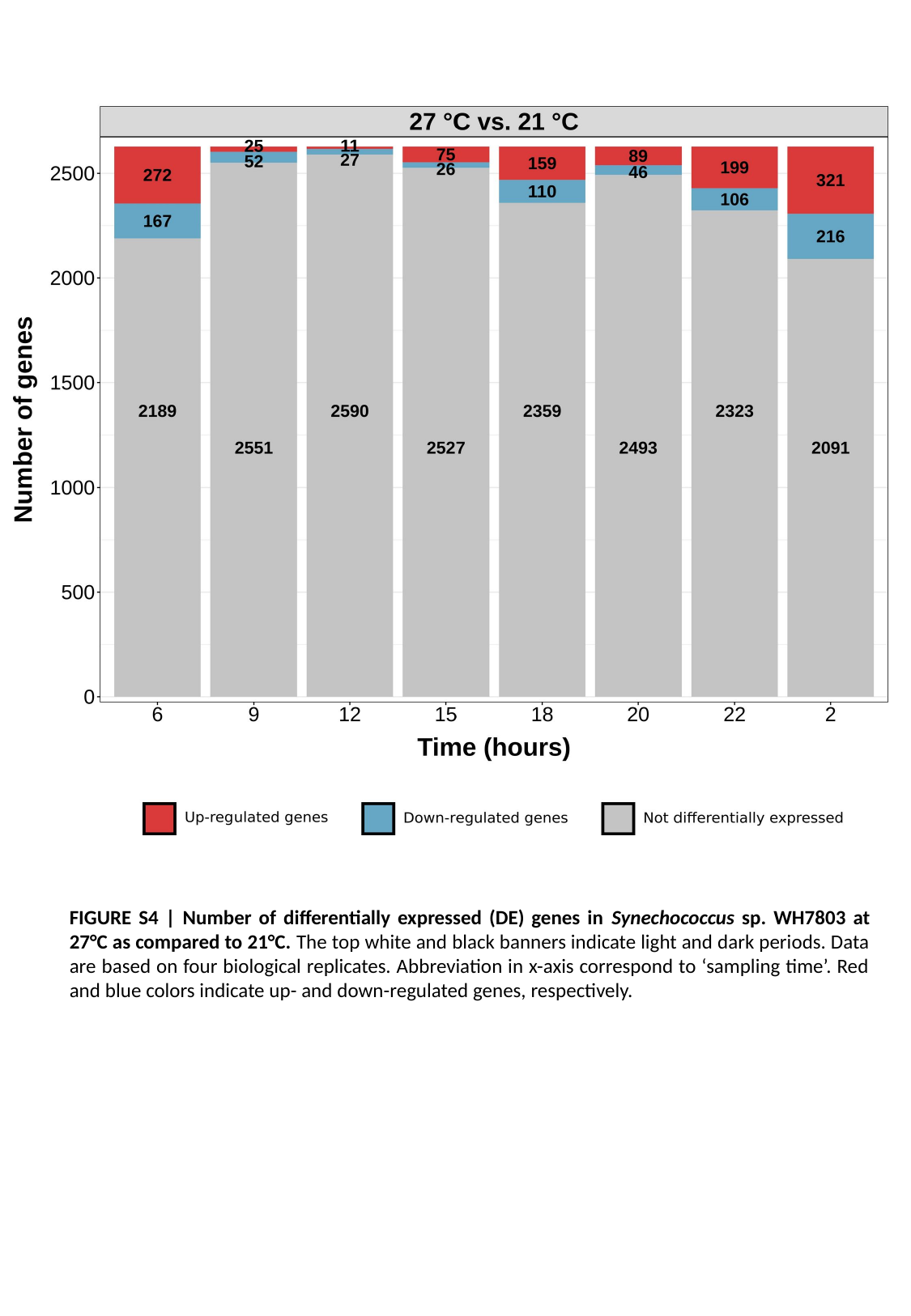

FIGURE S4 | Number of differentially expressed (DE) genes in Synechococcus sp. WH7803 at 27°C as compared to 21°C. The top white and black banners indicate light and dark periods. Data are based on four biological replicates. Abbreviation in x-axis correspond to ‘sampling time’. Red and blue colors indicate up- and down-regulated genes, respectively.

## Slide 5
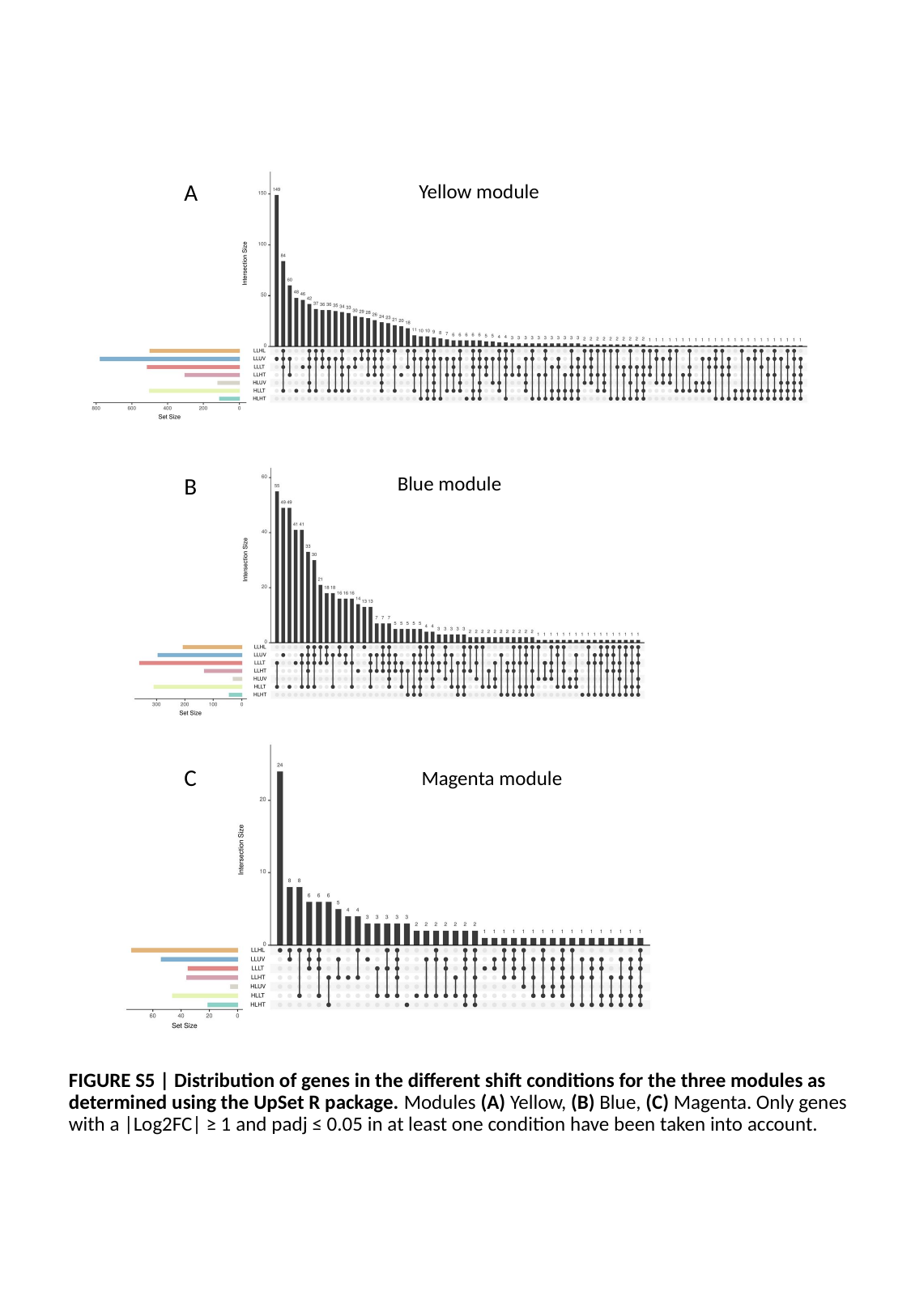

Yellow module
A
Blue module
B
C
Magenta module
FIGURE S5 | Distribution of genes in the different shift conditions for the three modules as determined using the UpSet R package. Modules (A) Yellow, (B) Blue, (C) Magenta. Only genes with a |Log2FC| ≥ 1 and padj ≤ 0.05 in at least one condition have been taken into account.

## Slide 6
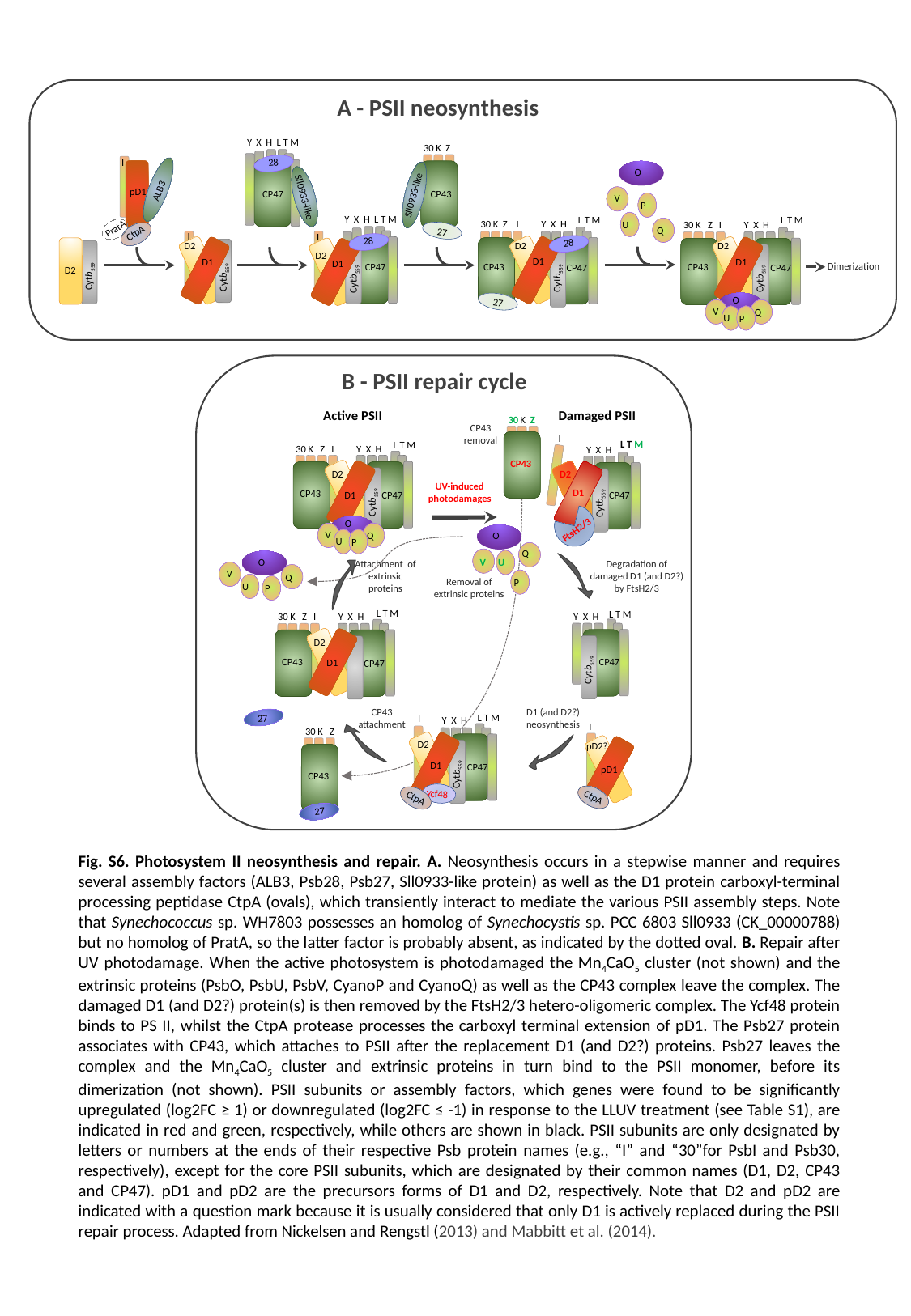

A - PSII neosynthesis
Y X H L T M
30 K Z
28
I
O
ALB3
I
pD1
CP47
CP43
Sll0933-like
Sll0933-like
V
P
Y X H L T M
L T M
L T M
30 K Z I Y X H
U
30 K Z I Y X H
PratA
Q
27
CtpA
I
I
28
28
D2
D2
D2
D2
D1
D1
D1
D1
Dimerization
CP47
CP43
CP43
CP47
CP47
D2
Cytb559
Cytb559
Cytb559
Cytb559
Cytb559
O
27
V
Q
U
P
B - PSII repair cycle
Active PSII
Damaged PSII
30 K Z
CP43 removal
I
L T M
L T M
30 K Z I Y X H
Y X H
CP43
D2
D2
UV-induced photodamages
D1
CP43
D1
CP47
CP47
Cytb559
Cytb559
O
FtsH2/3
V
Q
O
U
P
Q
V
U
O
Attachment of extrinsic proteins
Degradation of damaged D1 (and D2?)
by FtsH2/3
V
Q
Removal of extrinsic proteins
P
U
P
L T M
L T M
30 K Z I Y X H
Y X H
D2
CP43
CP47
D1
CP47
Cytb559
CP43 attachment
D1 (and D2?) neosynthesis
L T M
27
I
Y X H
I
30 K Z
D2
pD2?
D1
CP47
pD1
Cytb559
CP43
Ycf48
CtpA
CtpA
27
Fig. S6. Photosystem II neosynthesis and repair. A. Neosynthesis occurs in a stepwise manner and requires several assembly factors (ALB3, Psb28, Psb27, Sll0933-like protein) as well as the D1 protein carboxyl-terminal processing peptidase CtpA (ovals), which transiently interact to mediate the various PSII assembly steps. Note that Synechococcus sp. WH7803 possesses an homolog of Synechocystis sp. PCC 6803 Sll0933 (CK_00000788) but no homolog of PratA, so the latter factor is probably absent, as indicated by the dotted oval. B. Repair after UV photodamage. When the active photosystem is photodamaged the Mn4CaO5 cluster (not shown) and the extrinsic proteins (PsbO, PsbU, PsbV, CyanoP and CyanoQ) as well as the CP43 complex leave the complex. The damaged D1 (and D2?) protein(s) is then removed by the FtsH2/3 hetero-oligomeric complex. The Ycf48 protein binds to PS II, whilst the CtpA protease processes the carboxyl terminal extension of pD1. The Psb27 protein associates with CP43, which attaches to PSII after the replacement D1 (and D2?) proteins. Psb27 leaves the complex and the Mn4CaO5 cluster and extrinsic proteins in turn bind to the PSII monomer, before its dimerization (not shown). PSII subunits or assembly factors, which genes were found to be significantly upregulated (log2FC ≥ 1) or downregulated (log2FC ≤ -1) in response to the LLUV treatment (see Table S1), are indicated in red and green, respectively, while others are shown in black. PSII subunits are only designated by letters or numbers at the ends of their respective Psb protein names (e.g., “I” and “30”for PsbI and Psb30, respectively), except for the core PSII subunits, which are designated by their common names (D1, D2, CP43 and CP47). pD1 and pD2 are the precursors forms of D1 and D2, respectively. Note that D2 and pD2 are indicated with a question mark because it is usually considered that only D1 is actively replaced during the PSII repair process. Adapted from Nickelsen and Rengstl (2013) and Mabbitt et al. (2014).

## Slide 7
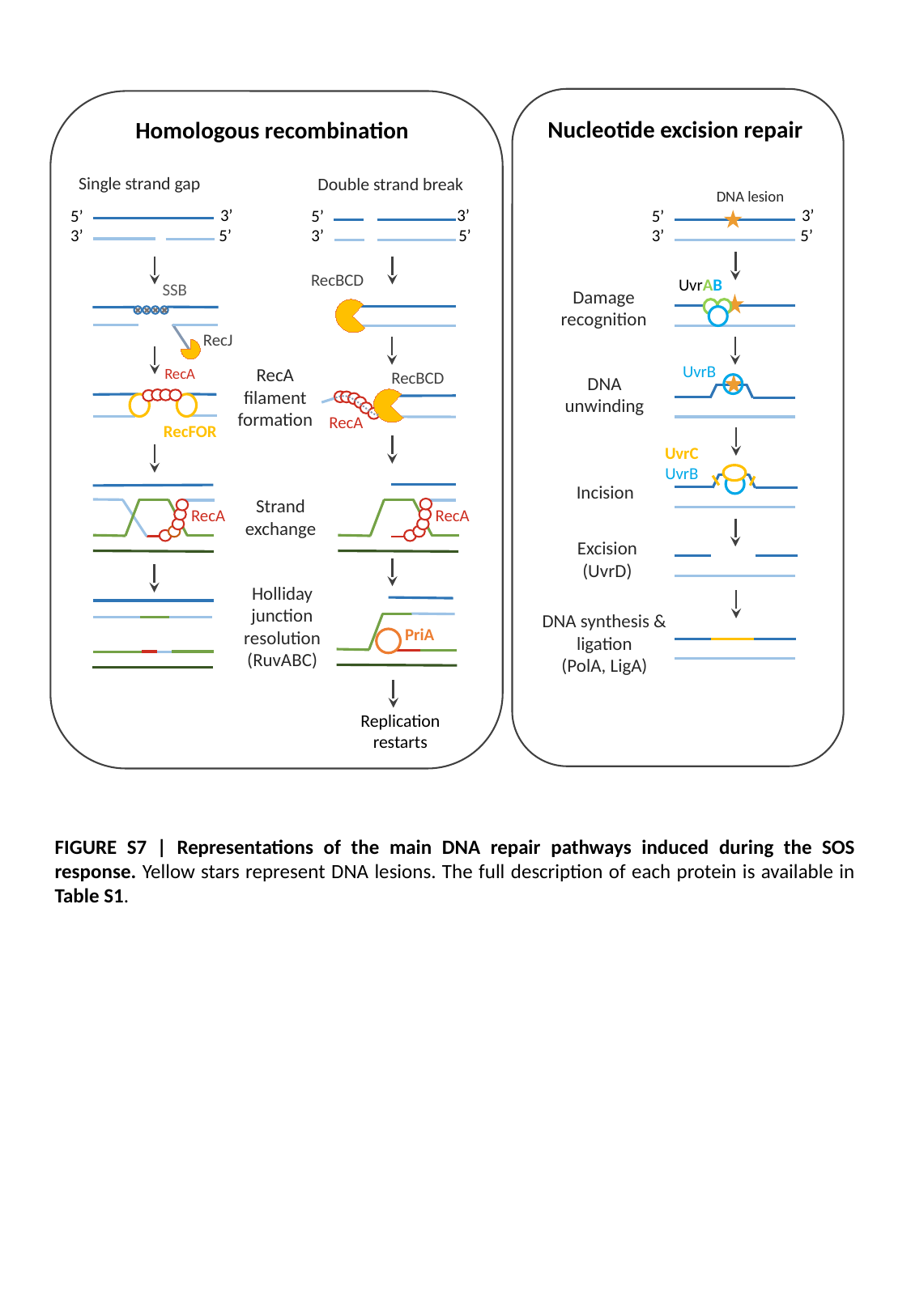

Nucleotide excision repair
Homologous recombination
Single strand gap
Double strand break
DNA lesion
3’
3’
3’
5’
5’
5’
5’
3’
3’
5’
5’
3’
RecBCD
UvrAB
SSB
Damage recognition
RecJ
UvrB
RecA filament formation
RecA
RecBCD
DNA unwinding
RecA
RecFOR
UvrC
UvrB
Incision
Strand exchange
RecA
RecA
Excision
(UvrD)
Holliday junction resolution
(RuvABC)
DNA synthesis & ligation
(PolA, LigA)
PriA
Replication restarts
FIGURE S7 | Representations of the main DNA repair pathways induced during the SOS response. Yellow stars represent DNA lesions. The full description of each protein is available in Table S1.

## Slide 8
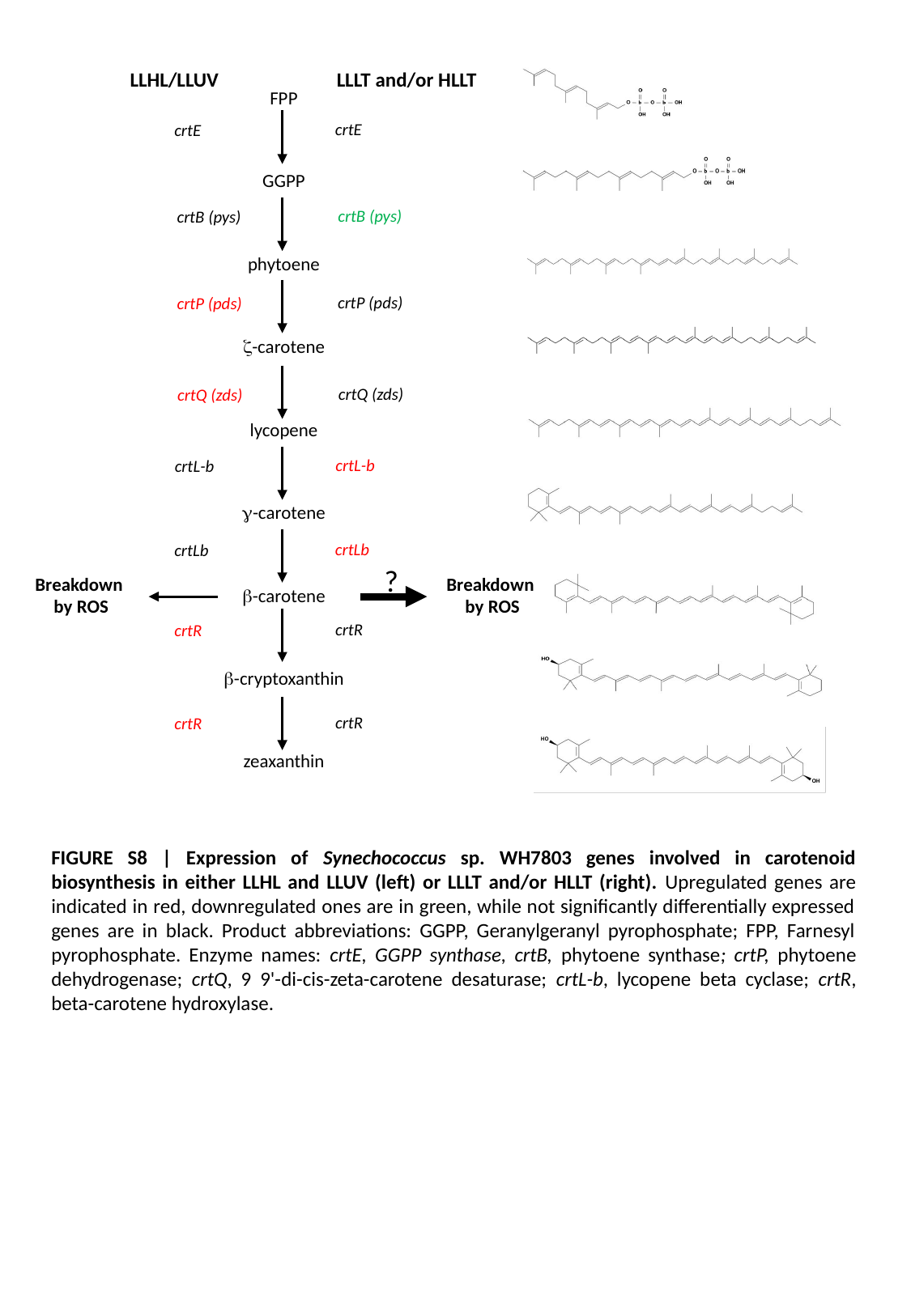

LLHL/LLUV
LLLT and/or HLLT
FPP
crtE
crtE
GGPP
crtB (pys)
crtB (pys)
phytoene
crtP (pds)
crtP (pds)
z-carotene
crtQ (zds)
crtQ (zds)
lycopene
crtL-b
crtL-b
g-carotene
crtLb
crtLb
?
Breakdown
by ROS
Breakdown
by ROS
b-carotene
crtR
crtR
b-cryptoxanthin
crtR
crtR
zeaxanthin
FIGURE S8 | Expression of Synechococcus sp. WH7803 genes involved in carotenoid biosynthesis in either LLHL and LLUV (left) or LLLT and/or HLLT (right). Upregulated genes are indicated in red, downregulated ones are in green, while not significantly differentially expressed genes are in black. Product abbreviations: GGPP, Geranylgeranyl pyrophosphate; FPP, Farnesyl pyrophosphate. Enzyme names: crtE, GGPP synthase, crtB, phytoene synthase; crtP, phytoene dehydrogenase; crtQ, 9 9'-di-cis-zeta-carotene desaturase; crtL-b, lycopene beta cyclase; crtR, beta-carotene hydroxylase.
